# YAP/TAZ drives Notch and angiogenesis mechanoregulation in silico

**DOI:** 10.1038/s41540-024-00444-3

**Published:** 2024-10-05

**Authors:** Margot Passier, Katie Bentley, Sandra Loerakker, Tommaso Ristori

**Affiliations:** 1 Department of Biomedical Engineering, Eindhoven University of Technology, Eindhoven, the Netherlands; 2 Institute for Complex Molecular Systems (ICMS), Eindhoven University of Technology, Eindhoven, the Netherlands; 3 The Francis Crick Institute, London, UK; 4 Department of Informatics, King’s College London, London, UK

**Keywords:** Numerical simulations, Biomedical engineering

## Abstract

Endothelial cells are key players in the cardiovascular system. Among other things, they are responsible for sprouting angiogenesis, the process of new blood vessel formation essential for both health and disease. Endothelial cells are strongly regulated by the juxtacrine signaling pathway Notch. Recent studies have shown that both Notch and angiogenesis are influenced by extracellular matrix stiffness; however, the underlying mechanisms are poorly understood. Here, we addressed this challenge by combining computational models of Notch signaling and YAP/TAZ, stiffness- and cytoskeleton-regulated mechanotransducers whose activity inhibits both Dll4 (Notch ligand) and LFng (Notch-Dll4 binding modulator). Our simulations successfully mimicked previous experiments, indicating that this YAP/TAZ-Notch crosstalk elucidates the Notch and angiogenesis mechanoresponse to stiffness. Additional simulations also identified possible strategies to control Notch activity and sprouting angiogenesis via cytoskeletal manipulations or spatial patterns of alternating stiffnesses. Our study thus inspires new experimental avenues and provides a promising modeling framework for further investigations into the role of Notch, YAP/TAZ, and mechanics in determining endothelial cell behavior during angiogenesis and similar processes.

## Introduction

Endothelial cells (ECs) lining blood vessels drive the formation of new blood vessels via a process called angiogenesis^1^ and regulate the permeability of the resulting vessels^2^. In case of dysregulated behavior, pathological conditions arise ranging from vascular malformations to leakage of the vessels^3^. Hence, identification of the determinants of endothelial cell behavior is of the utmost importance to understand cardiovascular diseases and to develop appropriate medical therapies. Recently, it has become apparent that one of those determinants is the stiffness of the extracellular matrix (ECM) surrounding the ECs^4–7^. However, the biological mechanisms driving stiffness-dependent EC behavior are still unclear. A better understanding of these regulatory mechanisms would benefit treatment of EC-associated diseases, as well as allow for the development of relatively large tissue engineered constructs, currently still hampered by their lack of physiological vascularization^8^.

EC fate is strongly influenced by Notch, a juxtacrine signaling pathway essential for the development of almost all tissues in the human body^9–11^. Through Notch signaling, many different functions are achieved and a plethora of regulatory mechanisms are in play to adapt the outcome towards different situations^11^. In human ECs, the Notch pathway mainly comprises two receptors (Notch1 and 4) and two ligands (Delta like4 -Dll4- and Jagged1)^12,13^. When a ligand on one cell binds to the Notch receptor on a neighboring EC, the receptor in the receiving cell is cleaved, releasing the Notch Intracellular Domain (NICD). This signaling fragment can then travel to the nucleus, where it affects transcription of Notch target genes^14^ which, in turn, contribute to the determination of EC phenotype and behavior. Such cell-to-cell communication via Notch enables the generation of patterns of cell phenotypes^15,16^ via either lateral inhibition, where a signal receiving cell hampers the ability of adjacent cells to attain the same cell fate, or lateral induction, in which instead cells stimulate their neighbors to take on the same fate^14^. Increasing evidence demonstrates that this process is mechanoresponsive^17^. Accordingly, recent studies have shown that Notch activity in ECs is strongly influenced by the ECM stiffness, showing increased Notch activity on softer 2D substrates compared to stiffer ones^6,18^. We hypothesized that this Notch mechanoresponse could be a fundamental determinant of EC function in response to ECM stiffness. However, the key players explaining this Notch mechanoresponse are currently unknown, which hinders the control of EC-related mechanisms.

One of the processes strongly regulated by Notch is angiogenesis^19^. At the onset of angiogenesis, by crosstalk with Vascular Endothelial Growth Factor (VEGF), Notch ensures the establishment of an alternating pattern of tip, leading migratory cells, and stalk, following, proliferating cells^20,21^. More specifically, when hypoxic tissues secrete pro-angiogenic VEGF, this can bind to the VEGF receptors (VEGFRs) on ECs^19^. Resulting from VEGFR activation, filopodia growth is stimulated^22^ further enhancing the VEGF sensing ability of this cell^22,23^. In addition, Dll4 expression is upregulated^24^. The upregulated Dll4 binds to and activates Notch1 (further referred to as Notch) on its neighbors, thereby regulating VEGFR, causing an overall decrease of the VEGF sensing ability of these cells^25^. So, while VEGFR activation stimulates ECs to increase filopodia formation and attain a tip migratory behavior^23^, Notch activation forces the neighboring ECs to retain a proliferative stalk phenotype^23,26^, thereby establishing a pattern of tip ECs alternated by stalk ECs^15,24,27,28^. After patterning, tip cells lead the formation of nascent sprouts while stalk cells proliferate to make up the lumen, until the sprouts fuse to already existing networks^19,23^. Recent studies have revealed that the sprouting process is affected by the ECM stiffness too; for relatively low stiffness regimes, the number of sprouts was observed to increase with ECM stiffness^4^. Given the role of Notch for the tip/stalk pattern formation and its sensitivity to stiffness, we hypothesize that Notch mechanoregulation might lie at the basis of this observed angiogenic response to stiffness. Unraveling the Notch mechanoresponse to stiffness might therefore clarify the dependence of angiogenesis on stiffness and provide novel tools to steer this process.

In addition to this, increasing evidence demonstrates that Notch is influenced by YAP/TAZ^18,29–31^, well-known mechano-sensitive transcriptional regulators whose location and activity are dictated by stiffness-sensitive cytoskeletal processes^32,33^. In particular, YAP/TAZ reside in the cytoplasm on softer substrates, whereas their nuclear presence increases with increasing substrate stiffness^34,35^. YAP/TAZ nuclearization is dependent on several cytoskeletal processes, with an event cascade starting from integrin binding to stress fiber formation and nuclear deformations^36–38^. Interestingly, experimental findings have uncovered that YAP/TAZ nuclearization in ECs downregulates the expression of Notch ligand Dll4^18,29,31^. While this can at least partly explain the Notch response to stiffness for confluent ECs^6,18^, it cannot lead to the previously observed Notch mechanoresponse for single ECs exposed to external Dll4^6^, which is in theory unaffected by expression of intracellular Dll4. Another recent study showed that nuclear YAP/TAZ also inhibits Lunatic Fringe (LFng)^29^, an enzyme that causes glycosylation of Notch and consequentially higher Notch-Dll4 binding^39,40^. This could potentially explain single cell behavior, although the exact implications are still largely unknown. Here, we hypothesize that YAP/TAZ inhibition of both Dll4 and LFng are key factors for the Notch and angiogenic mechanoresponse to stiffness. To test this hypothesis, we developed a computational framework in which we coupled the YAP/TAZ and Notch cascades via inhibitory interactions between YAP/TAZ and Dll4 as well as LFng. The model was adopted to unravel the mechanisms of Notch and angiogenesis mechanoregulation as well as to explore novel methods of guiding angiogenesis.

Computational models have already been successful in the past to unravel the mechanisms of YAP/TAZ, Notch and angiogenesis^17,41,42^. The mechanoregulated cytoskeletal remodeling leading to YAP/TAZ nuclearization in response to stiffness has been previously modeled via ordinary differential equations (ODE)^37^, and then adopted in different settings^43–45^. Computational models directly imposing a Notch mechanoresponse have been developed to investigate the mechanisms of vessel homeostasis and adaptation to changing mechanical stimuli^46–49^. In the context of angiogenesis, computational models of Notch signaling have been successful in elucidating that Notch is a temporal regulator of the tip/stalk patterning rate and filopodia activity, which influence the resulting vascular topology^50,51^. Combined with experiments, these models have indicated that LFng affects angiogenesis by influencing the Notch and tip/stalk patterning temporal dynamics^27,52^. Therefore, we hypothesize that the influence of YAP/TAZ on Dll4 and LFng might contribute not only to Notch activity regulation in ECs in response to stiffness, but also to regulation of the timing of tip/stalk pattern selection and, as result, also the spacing of branches and therefore the network topology resulting from sprouting angiogenesis. In the present study, we built on previous computational approaches to validate these hypotheses. In particular, we developed a novel computational framework by coupling previous ODE models focused on the dynamics of the VEGF-Notch crosstalk^27,52^ and the ECM- and cytoskeleton-mediated YAP/TAZ pathway^37^, via inhibitory relations between YAP/TAZ and the Notch-pathway elements. Upon testing our hypotheses and providing a possible explanation for Notch mechanoregulation and, consequentially, the angiogenic mechanoresponse, we adopted the model as a tool to investigate possible interventions to control Notch activity and angiogenesis. Specifically, we studied knock down and upregulation of cytoskeletal elements as well as several heterogeneous stiffness patterns. The developed model and newly obtained insights open the door to new experiments and applications in mechanobiology and tissue engineering.

## Results

### Simulating YAP/TAZ nuclearization and Dll4 stiffness dependence in ECs

**Fig. 1:**
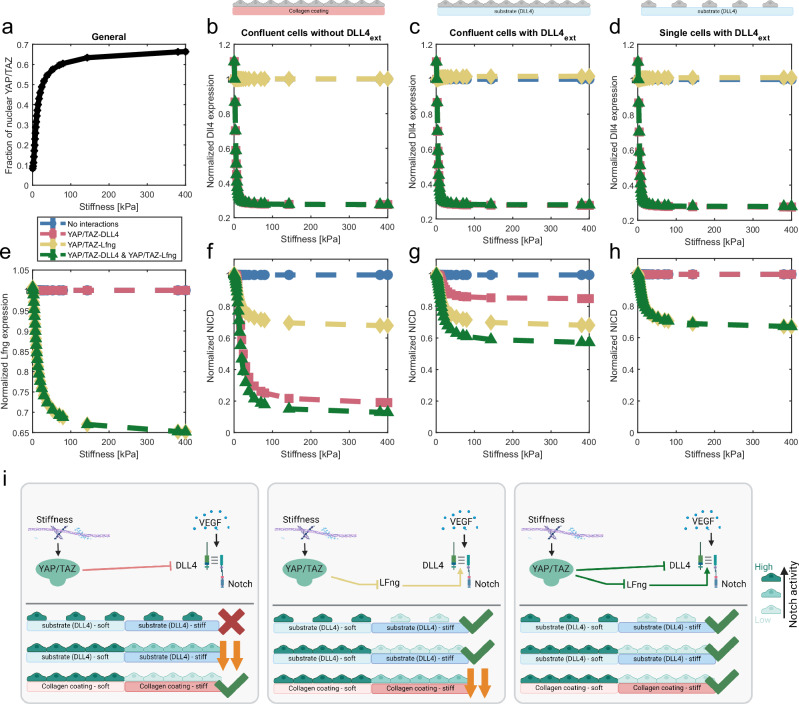
YAP/TAZ inhibition of Dll4 and LFng determines the Notch mechanoresponse to stiffness in silico. For **a**–**g**, simulations were repeated 25 times. Here, median and interquartile range (IQR) are reported, although the IQR is not clearly visible, due to variations being extremely low. For **b**–**h**, blue represents simulations without YAP/TAZ-Notch interactions, red represents inclusion of YAP/TAZ inhibition of Dll4, yellow indicates LFng inhibition by YAP/TAZ and green indicates the inclusion of both interactions. **a** For all simulated cases, the YAP/TAZ nuclear fraction increases for increasing stiffness, irrespective of the Notch-YAP/TAZ crosstalk, while LFng decreases with stiffness only when the YAP/TAZ-LFng interaction is accounted for, **e**. Dll4 and NICD trends changed for confluent cell layers (**b**, **f**), confluent cell layers exposed to Dll4 coating (**c**, **g**) and single cells on a Dll4 coating (**d**, **h**). Dll4 expression decreases with increasing stiffness only when the YAP/TAZ-Dll4 interaction is included (**b**–**d**). For confluent cells (**f**, **g**), NICD decreases for increasing stiffness, when either YAP/TAZ-LFng or YAP/TAZ-Dll4 interactions are included, while inclusion of YAP/TAZ-LFng is essential to observe a decrease for single cells (**h**). These findings were schematically summarized in **i**. All schematics were created using BioRender.

First, we tuned the parameters of the YAP/TAZ model to mimic ECs, as the original YAP/TAZ model^37^ mimicked the effects that ECM stiffness had on cytoskeletal components of cells other than ECs, resulting in YAP/TAZ nuclearization for stiffnesses higher than 10 kPa. Since previous experiments with ECs have shown a sharp increase of YAP/TAZ nuclearization already going from 0.5 kPa to 1.5 kPa^6,53^, the parameters of the YAP/TAZ model were calibrated to match this endothelial YAP/TAZ response. In particular, given their large effect on the model outcome and direct interaction with environmental stiffness^37^, we calibrated the parameters related to focal adhesion kinase (FAK), such that the model simulations showed a sharp increase of nuclear YAP/TAZ already at relatively low stiffnesses (0.5–1.5 kPa, Fig. 1a), further increasing for 70 kPa and ultimately approaching a plateau for high stiffnesses ( ~ 400 kPa. Figure 1a, see Methods for more details). This YAP/TAZ sensitivity for relatively low stiffnesses is consistent with experimental data not only on YAP/TAZ nuclearization^6^, but also on YAP target genes^18^. The range of stiffnesses (0.5 kPa–400 kPa) was based on previous experiments^6^ indicating that the most significant response to stiffness in terms of YAP/TAZ nuclearization and Notch activity occurs within this range. The stiffness increments were determined by taking 20 equal steps in terms of nuclear YAP/TAZ, yielding 20 stiffness values, as further described in the Methods and Fig. S1.

Matsuo et al.^18^ also observed decreased Dll4 protein expression in response to stiffness-induced YAP/TAZ nuclearization, consistent with previously shown effects of YAP/TAZ on Dll4^29^. To account for this effect, we here coupled the YAP/TAZ and VEGF-Notch model by implementing an inhibitory relation between YAP/TAZ activity and Dll4 expression, with parameters calibrated against previous experiments^18^. The computational model including this interaction recapitulates the initial sharp decrease in Dll4 expression for increasing stiffness and reduced sensitivity to higher stiffnesses^18^ (Fig. 1b).

### YAP/TAZ-Dll4 and YAP/TAZ-LFng interactions can explain Notch mechanoregulation

With the coupled model, we conducted simulations of Notch activity in confluent and single ECs cultured on 2D substrates with varying stiffnesses, with and without Dll4 coating (Fig. 1), thereby mimicking previous experiments^6,18^ to investigate the underlying mechanisms of Notch mechanoregulation. For all these simulations (Fig. 1), in agreement with the lack of VEGF stimulation, no tip/stalk patterning was predicted, with homogeneous levels of Dll4 expression and NICD across cells, which rapidly reached a constant level over time (see Fig. S2 for a representative case). Given this homogeneity across cells, the average values depicted in Fig. 1 represent the values in each individual cell. Consistent with the central role of Dll4 expression for Notch activity in endothelial cells^28^ and with the results of the simulated experiments^6,18^, our model with stiffness-regulated Dll4 expression predicted a reduction of Notch activity for increasing stiffnesses in confluent ECs, both without (Fig. 1f) and with Dll4 coating (Fig. 1g). However, in contrast with experiments^6^, the model coupled solely through the YAP/TAZ–Dll4 inhibition showed no effect of stiffness on Notch activity for single cells on a Dll4-coated substrate (Fig. 1h). This conclusion was not dependent on the parameters chosen, as shown via a sensitivity analysis (Fig. S3). The lack of stiffness effects for this model version can be explained by YAP/TAZ only affecting the amount of intracellular Dll4 in this version of the model, which has no role in Notch activation in the single cell experiments, where cells are rather activated by the (untargeted) Dll4 coating. Therefore, our model with only the YAP/TAZ-Dll4 interaction can explain Notch regulation by stiffness for a confluent cell layer, but not for single cells (Fig. 1i, left panel).

The stiffness regulation of Notch activity occurring even in single cells indicates that stiffness affects not only the signaling cell, by regulating Dll4 expression, but also the receiving cells. Given the effects of Fringes on the Notch receptors^54^, we hypothesized that the observed behavior can be explained by a stiffness-mediated regulation of Notch ligand-receptor binding via the known binding affinity modulator LFng^39,40^. To test our hypothesis, we implemented a second inhibitory interaction in which stiffness modulates LFng expression through YAP/TAZ, backed up by experimental findings of LFng upregulation resulting from YAP/TAZ knock down^29^. Our computational results confirmed that downregulation of LFng by YAP/TAZ nuclearization can cause a stiffness-mediated downregulation of Notch activity in single ECs stimulated with a Dll4 coating (Fig. 1h), without having a great impact on Dll4 expression (Fig. 1d). Inclusion of both Dll4 and LFng downregulation by YAP/TAZ can thus replicate all previous observations (Fig. 1i) and leads to an even bigger, multiplicative decrease in Notch activity for the confluent cases (Fig. 1f, g), while having a minor impact on Dll4 expression (Fig. 1b, c). In summary, the simulations suggest that LFng regulation by stiffness via YAP/TAZ is a fundamental mechanism to explain the mechanisms of Notch stiffness-regulation. Moreover, we observed that the YAP/TAZ-LFng interaction has a relatively larger contribution in settings including a Dll4 coating, whereas the YAP/TAZ- Dll4 interaction has a larger effect in settings without coating.

### Manipulation of cytoskeletal elements might rescue Notch activity on stiff substrates

The developed coupling between the cytoskeleton-mediated YAP/TAZ model^37^ and the Notch model^27^ enabled us to simulate and predict the effects that (perturbations of) cytoskeletal elements might have on Dll4 and LFng expression and, ultimately, on Notch activity for different stiffnesses. This interplay could regulate several biological processes, given the relevance of Notch activity for several phenomena^13,55^. Therefore, we conducted simulations with a focus on Notch activity using an activating coating of Dll4, in which we investigated up- and downregulation of cytoskeletal elements. The perturbations corresponded to downregulation (by commonly adopted inhibitors) of Myosin (Blebbistatin)^56^, ROCK (Y-27362)^57^, F-actin (Latrunculin)^58^ or upregulation (by a common activator) of RhoA (LPA)^59^. For completeness, we also simulated the opposite effects of these inhibitors and activators. Moreover, perturbations of Cofilin were investigated because of its downregulatory effect on YAP/TAZ compared to the other perturbed elements (Myosin, ROCK, RhoA and F-actin), which instead upregulate YAP/TAZ. In what follows, “cytoskeletal elements” thus indicate the group of proteins composed of Myosin, ROCK, F-actin and RhoA, which we separate from Cofilin, due to its opposing effect on YAP/TAZ.

**Fig. 2:**
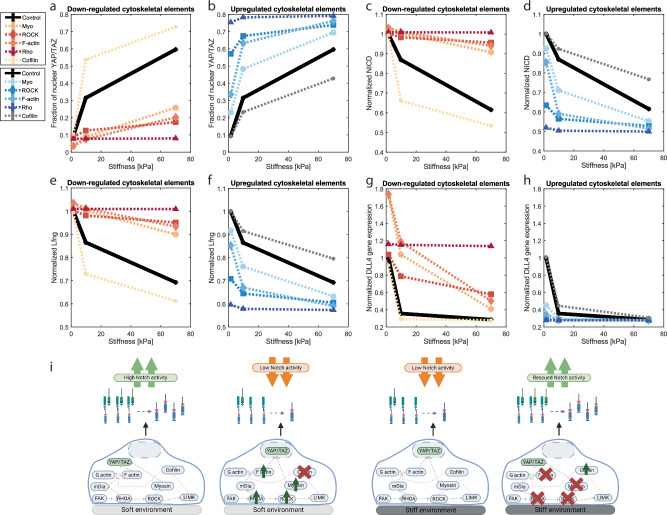
Cytoskeletal manipulations can rescue or inhibit Notch activity in silico. **a**–**h** Simulation of confluent EC layers exposed to a Dll4 coating and different stiffness levels, with upregulation (blue) or downregulation (orange) of cytoskeletal elements (Myosin, ROCK, F-actin, Rho, Cofilin) compared to control (black). All simulations were repeated 25 times, for which here, median and IQR are shown. Simulations were performed to investigate, for cytoskeletal perturbations, the effects of increasing stiffness on YAP/TAZ nuclear fraction (**a**, **b**), NICD (**c**, **d**), normalized expression of LFng (**e**, **f**) and normalized expression of DLL4 (**g**, **h**). A schematic representation of the simulation results is visualized in **i**, which was created using BioRender.

In agreement with previous simulations with the YAP/TAZ model^37^, knock down of the cytoskeletal elements resulted in decreased nuclear YAP/TAZ fractions, especially for higher stiffnesses (Fig. 2a). From a mechanistic point of view, this occurs because the chain of reactions leading to YAP/TAZ nuclearization is disrupted by the cytoskeletal interventions; as a result, YAP/TAZ (partially) loses its mechanosensitivity and its localization is similar to that on soft substrates; most of it resides in the cytoplasm. Accordingly, Dll4 and LFng expression remain relatively unaffected by stiffness for these knock-down cases and are no longer (or to a lesser extent) downregulated for high stiffnesses (Fig. 2e, g). The relatively high expression of Dll4 and LFng predicted also for stiff substrates leads to consequentially high Dll4-Notch binding, and sustained Notch activity. Notch activity can thus be rescued by cytoskeletal downregulation on stiff environments (Fig. 2c). The opposite trends are observed for upregulation of the cytoskeletal elements, which causes an increased stiffness-mediated YAP/TAZ activity (Fig. 2b) and downregulatory effect on Dll4, LFng and, consequentially, Notch activity (Fig. 2d, f, h). Due to the inhibitory relationship between Cofilin and YAP/TAZ, Cofilin perturbations show the opposite trends, with Cofilin upregulation leading to rescued Dll4 and LFng expressions as well as Notch activity on stiff substrates, and vice versa for Cofilin downregulation. In summary, the simulations suggest that the stiffness-mediated downregulation of Dll4, LFng, and Notch activity can be counteracted or exacerbated by perturbing cytoskeletal elements, such as Myosin, in turn affecting YAP/TAZ nuclearization (Fig. 2i).

### Stiffness induces angiogenic tip-cell selection and a biphasic trend for tip/stalk patterning rates

Previous studies have shown that angiogenic sprouting is affected by the stiffness of the extracellular matrix^4^. Given that Notch activity is one of the main regulators of angiogenic patterning and sprouting^26^, we investigated whether the angiogenic patterning response to stiffness can be explained by YAP/TAZ downregulation of Dll4 and LFng. More specifically, we focused our attention on the effects on the temporal dynamics of tip/stalk patterning at the onset of sprouting, previously shown to influence the network topology of the final vasculature^50^. In particular, while relatively fast patterning leads to physiological angiogenesis, relatively slow patterning can lead to either sparse network formation or dense malformed networks^50^, resulting from generally low filopodia activity, or generally high filopodia activity respectively. Previous experiments and simulations highlighted LFng as a regulator of tip/stalk selection timing and network formation^27,52^. In combination with the central role of Dll4 for angiogenesis^28^, we expect that stiffness regulates the rate of patterning and network formation by regulating Dll4 and LFng via YAP/TAZ stiffness sensitivity. To test this hypothesis, we simulated patterning of cells by exposing them to VEGF without a Notch-activating Dll4 coating, including YAP/TAZ interactions with either Dll4, LFng, both or neither of the two. Most of the predictions yielded either uniform or salt-and-pepper patterns of tip/stalk cells, although occasional disordered patterns with two adjacent tip cells or two adjacent stalk cells were predicted (Fig. S4), similar to previous in silico studies^60,61^.

In our simulations, consistent with previous studies^52,62,63^, a physiological pattern was considered to be achieved when cells exhibited a set of phenotypes consisting of at least 40% tip cells, without any adjacent tip cells. To obtain a proxy for the rate of tip/stalk pattern formation, as in Ristori et al.^52^, the value of 24 h available for cells to pattern upon VEGF exposure was divided by the earliest timepoint for which a physiological tip/stalk pattern was observed (${t}_{p}$) : $\frac{24[h]}{{t}_{p}[h]}$. Lastly, to increase readability, we only show the results corresponding to the subset of stiffnesses needed to portray the full trend.

**Fig. 3:**
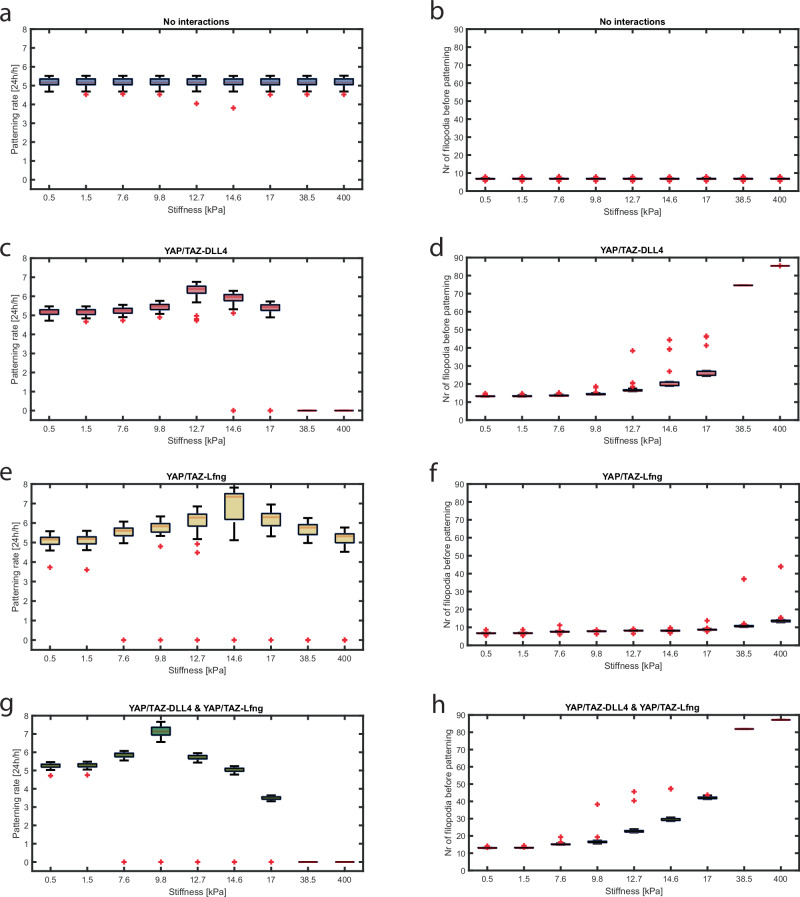
Stiffness influences the temporal dynamics of tip/stalk pattern formation and filopodia activity in silico. Boxplots of the systematic simulations investigating the effect of YAP/TAZ-Notch interactions on patterning rate (**a**, **c**, **e**, **g**) and filopodia activity (**b**, **d**, **f**, **h**). Stiffness did not have any effect without the implementation of YAP/TAZ-Notch interactions (**a**, **b**). With only the YAP/TAZ-Dll4 interaction (**c**, **d**), only the YAP/TAZ-LFng interaction (**e**, **f**) or both interactions (**g**, **h**) a biphasic response in terms of patterning rate and a general increase in filopodia activity to increasing stiffness was observed. All simulations were repeated 25 times, and the associated data are represented such that the boxes depict quartiles 2 and 3, the red line inside the box depicts the median, the whiskers indicate the min- and maximum and the red plusses indicate the outliers. The patterning rate was approximated by dividing 24 h by the patterning time; filopodia activity was determined by averaging the amount of filopodia in the timepoints before patterning occurred. The subset of stiffnesses was chosen to improve visibility while still accurately portraying the whole trend.

As expected, no effects of stiffness on patterning, filopodia activity, or the number of tip cells were observed when no hypothesized interactions between YAP/TAZ and the Notch pathway were included (Fig. 3a, b, and S5). Upon implementation of any of the YAP/TAZ-Notch inhibitory interactions however, the patterning rates showed a biphasic response to increases in stiffness, as well as increased filopodia activity before tip/stalk patterning, and increased percentages of tip cells (Fig. 3c–h and S5 b-d). This resulted from generally decreasing Notch activity for increasing stiffness. For low stiffnesses, there is little nuclear YAP/TAZ and consequentially little inhibition of Dll4 and LFng, yielding high Notch activity, high Notch target gene activity (HE) and, as a result, high lateral inhibition of VEGFR. This slows down filopodia formation (indicated by low filopodia activity for low stiffnesses, Fig. 3d, f, h) and consequently slows establishment of the tip cell phenotype and resulting patterning (Fig. 3c, e, g). We observed increasing patterning rates until reaching a peak patterning rate, with increases in substrate stiffness from 0.5 kPa up to 10–15 kPa depending on the included interactions, in agreement with previous experiments^4^. In this stiffness range, patterns containing two adjacent stalk cells sporadically occurred (see Fig. S4, a–c for an example). Above this threshold, patterning rates decreased as a function of substrate stiffness, due to the YAP/TAZ mediated decrease of Notch activity and extremely little lateral VEGFR inhibition left. As a result, all cells exhibited many filopodia (Fig. 3d, f, h) and took a long time to inhibit VEGFR and filopodia formation in neighboring cells: hence, pattern formation through the establishment of stalk cells slowed down (Fig. 3c, e, g). The location of this threshold indicating a shift from increasing towards decreasing patterning rates differed for each set of included YAP/TAZ interactions, going from a relatively higher threshold stiffness value for the simulations with solely the YAP/TAZ-LFng interaction (Fig. 3e), to a lower threshold stiffness value observed when both interactions were present (Fig. 3g). This effect stems from the relatively smaller contribution of YAP/TAZ-LFng compared to YAP/TAZ-Dll4 towards decreasing Notch activity for increasing stiffnesses in confluent settings without a Dll4 coating (Fig. 1f). Consistent with the stiffness-induced decrease in Notch activity, simulations with relatively high stiffnesses sporadically predicted unphysiological patterns with two adjacent tip cells (see Fig. S4, d-f for an example).

As an additional indication of the fact that the YAP/TAZ-Dll4 interaction has a major impact on angiogenesis, when such assumption is included in the model, it was predicted that cells cannot pattern, all become tip cells, for relatively high stiffnesses (38.5 kPa and upwards, Fig. 3c, g). This stems from high nuclear YAP/TAZ, yielding much Dll4 and LFng inhibition, resulting in very low Notch activity. As such, cells are unable to perform lateral inhibition and all cells become tip cells, exhibiting many filopodia (38.5 kPa and upwards, Fig. 3d, h). This effect is not observed when YAP/TAZ is only affecting LFng (Fig. 3e, f), consistent with our previous findings indicating a relatively smaller effect on Notch activity of YAP/TAZ mediated inhibition of LFng compared to YAP/TAZ inhibition of Dll4 in confluent cells without a Dll4 coating (Fig. 1f).

**Fig. 4:**
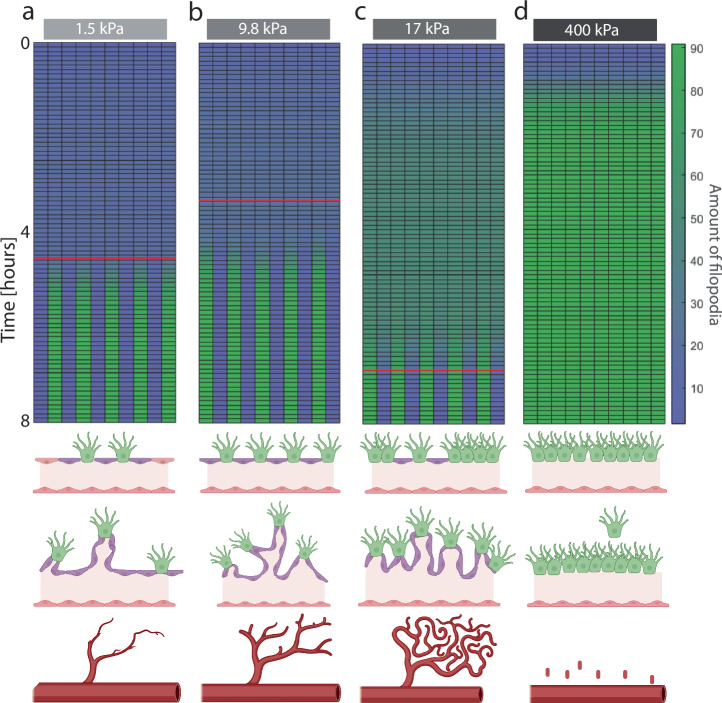
Representative simulations (top) of the time course of filopodia activity for four stiffnesses, depicting distinct patterning behaviors in the biphasic patterning response, with relative interpretations for vascular network formation (bottom^50^). The color scheme is representative of the cellular filopodia activity, tip cells indicated with bright green and stalk cells indicated in purple. **a** Relatively low stiffness (1.5 kPa) causes slow patterning, low filopodia activity and therefore sparse network formation. **b** Stiffness equal to 9.8 kPa induces fast patterning and intermediate filopodia activity, resulting in physiological network formation. **c** Relatively high stiffness (17 kPa) causes slow patterning but with high filopodia activity pre-patterning and, therefore, dense and malformed network formation. **d** Very high stiffness levels (400 kPa) do not induce tip/stalk patterning and induce generally high filopodia activity. The red lines indicate the timepoint of pattern formation. The schematics were created using BioRender.

In summary, for increasing stiffness, we found a biphasic response of patterning rates, as well as a generally increasing trend of filopodia formation. These two trends can be correlated to the different categories of patterning suggested by Bentley et al.^50^. For relatively low stiffnesses, cells pattern relatively slowly and present relatively low filopodia activity, which leads to them forming a sparse network (Fig. 4a). At peak patterning rate, cells pattern fast and present a moderate filopodia activity, leading to dense network formation (Fig. 4b). At slightly higher stiffnesses, cells pattern slowly, but with many cells prematurely exhibiting the tip cell phenotype, yielding dense malformed networks (Fig. 4c). At high stiffnesses, finally, cells cease to pattern and we observe an abundance of tip cells, which might lead to single cell migration (Fig. 4d)^64^. Consistent with previous experimental literature^26,28^, and similar to the alternating levels of filopodia for tip and stalk cells (Fig. 4a–c), we observed a pattern of relatively high Dll4 and low NICD for tip cells, alternated with low Dll4 and high NICD for stalk cells, respectively (Fig. S6).

### Manipulation of cytoskeletal elements can rescue pattern formation on stiff substrates

**Fig. 5:**
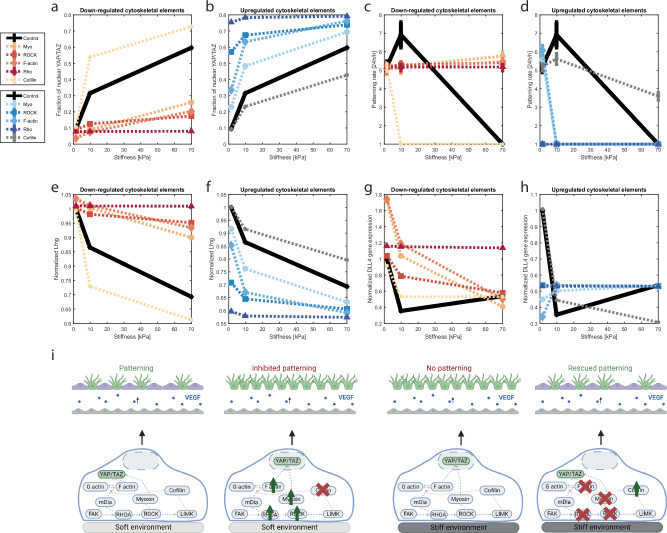
Cytoskeletal perturbations can rescue or diminish patterning in silico. **a**–**h** Simulation of confluent EC layers exposed to VEGF for 24 h to instigate patterning, after a 24 h acclimatization period. In these simulations, upregulation (blue) and downregulation (orange) of cytoskeletal elements (Myosin, ROCK, F-actin, Rho, Cofilin) was simulated and compared to control (black). Simulations were conducted 50 times and median values with IQR are shown here. The simulations were used to investigate: YAP/TAZ nuclear fractions (**a, b**), patterning rates (**c**, **d**), normalized expression of LFng (**e, f**) and normalized expression of Dll4 (**g, h**). Representative stiffnesses were chosen to visualize the response for: low stiffnesses; the intermediate stiffness corresponding to peak patterning rate; and high stiffness, for which patterning would not occur with the original parameters. A schematic representation of the simulation results is visualized in **i**, which was created using BioRender.

Given the predicted influence of cytoskeletal element perturbations on Notch activity (Fig. 2), we next investigated their possible effect on the tip/stalk patterning at the onset of angiogenesis. To this end, we simulated patterning of cells by exposing them to VEGF, without a Notch-activating Dll4 coating. Identical to the cellular behavior on a Dll4 coating without VEGF, in Fig. 2a, Fig. 5a shows decreased nuclear YAP/TAZ fractions upon knock down of cytoskeletal elements, with an inverse effect for Cofilin. Additionally, we observed a larger effect the more upstream the knocked-down cytoskeletal elements were. In terms of Notch components, consistent with previous simulations (Fig. 2), the LFng and Dll4 expression increase or decrease upon knock down or upregulation of cytoskeletal elements, respectively (Fig. 5e–h), although the control for Dll4 expression changes slightly (compared to Fig. 2g, h), due to ceased patterning of cells for the stiffest datapoint (Fig. 5g, h).

Analogous to knock down of cytoskeletal elements causing loss of stiffness-mediated Notch downregulation (Fig. 2), we observe decreased downregulation of patterning rates for increased stiffnesses and cytoskeletal knock down. In that case, Notch and Dll4 continue to bind even for high stiffnesses, as a consequence of rescued Dll4 and LFng expression, which also rescues tip/stalk patterning (Fig. 5c). The opposite can be observed for upregulation of cytoskeletal elements in cells surrounded by environments with low stiffness, which causes loss of tip/stalk patterning (Fig. 5d). Again, the trends for Cofilin are exactly opposite to those of the previously mentioned cytoskeletal elements. In conclusion, knock down of cytoskeletal elements can rescue patterning at higher stiffnesses, while upregulation of cytoskeletal elements can prevent patterning at low stiffnesses (Fig. 5i).

### Substrates with heterogeneous stiffnesses can hugely increase tip/stalk patterning rates and partially rescue patterning on stiffer substrates

**Fig. 6:**
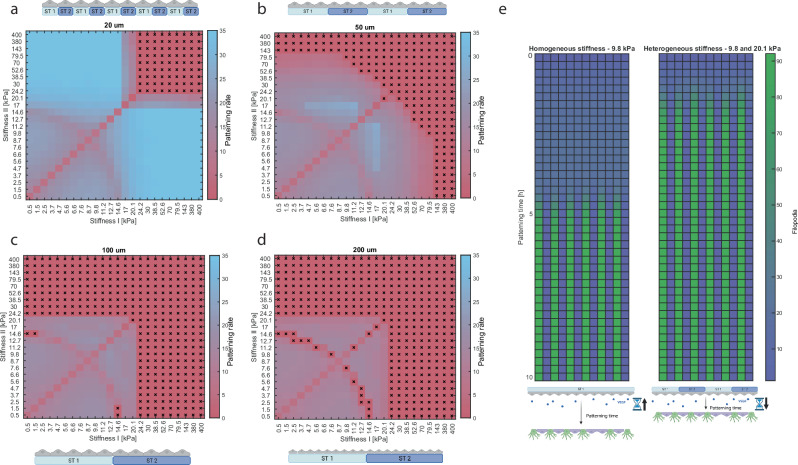
Heterogeneous stiffness patterns can accelerate patterning in silico. All combinations of the stiffnesses visualized in Fig. S1 were simulated and repeated 25 times, for which the medians are depicted in the colorplots. The colorplot indicates the patterning rate for each stiffness combination, for four different sizes of stiffness patterns (**a**–**d**). For all cases, the cells were exposed to alternating patches of stiffness I (x-axis) and stiffness II (y-axis). **a** Stiffness patterns of 20 um. **b** Stiffness patterns of 50 um. To allow for equal numbers of stiffness I and II patches, the number of simulated cells was increased here (see schematic above the colorplot). **c** Stiffness patterns of 100 um. **d** Stiffness patterns of 200 um, for which the row of simulated cells was further extended (see schematic below the colorplot). For **a**–**d** squares marked with an ‘x’ indicate cases that resulted in non-patterning (at least for more than 50% of the performed simulations). **e** A representative case from colorplot **b**, to show the difference in patterning speed between heterogeneous stiffness exposure (left) and heterogeneous stiffness exposure (right). All schematics were created using BioRender.

The simulations described in the previous sections were all performed for homogenous stiffnesses. Previously, we investigated the effects of heterogeneous patterns of Dll4 on spatial dynamics of endothelial sprouting^65^. Inspired by those findings and by the effect of stiffness on Dll4 expression (Fig. 1b–d), we hypothesized that alternating linear patterns of stiffness, such as the ones engineered by Jorba et al.^66^, might influence angiogenic sprout formation by kickstarting and thus accelerating patterning. To confirm this hypothesis, we simulated cells seeded on patches of alternating stiffness (labeled here with #I and #II), systematically varying their size and stiffness values (Fig. 6). The diagonals of Fig. 6a–d yield patterning rates of approximately 3–8 [24 h/h] (similar to Fig. 3g). Upon exposure to heterogeneous stiffness patterns, patterning rates increase tremendously (Fig. 6e). In case of two adjacent cells being exposed to different stiffnesses, this creates an initial difference in their Notch activity, ability for lateral inhibition and thus VEGF sensation. This initial difference kickstarts patterning, as cells no longer endure a (longer) period of almost equal behavior prior to patterning. This is particularly evident for single cell-sized patterns, for which the patterning rate can reach up to five times the patterning rate of its homogeneous analog (Fig. 6a). However, the larger the regions of equal stiffness, the smaller the increase in patterning rate (Fig. 6a, d).

Interestingly, larger differences between stiffness #I and stiffness #II do not always result in larger increases in patterning rate. Our results suggest that one stiffness value being close to the threshold (20.1 kPa) above which no patterning occurs for homogeneous stiffness, yields the highest patterning rates (Fig. 6b–d). This is because cells exposed to stiffnesses close to 20.1 kPa are very easily outcompeted. Moreover, all heatmaps show an arch corresponding to cases of slower patterning. This results from similar patterning rates for the two distinct stiffnesses (#I and #II), but through two different processes: one low stiffness value, yielding high Notch activity, and one high stiffness value, exhibiting little Notch activity, resulting in similar patterning rates. This similarity disrupts the tip/stalk pattern formation for heterogeneous cases. Finally, it can be observed that cells continue to pattern for stiffnesses above the homogenous threshold of 20.1 kPa (Fig. 6a, b), although this effect is limited and quickly fades upon increasing sizes of the stiffness regions for #I and #II (Fig. 6c, d). To conclude, the simulations suggest that heterogeneous stiffness patterns can accelerate the tip/stalk patterning at the onset of angiogenesis, with the scale of the increase depending on the size of the different stiffness regions.

## Discussion

Elucidation of the mechanisms determining EC behavior is key to better understanding cardiovascular pathologies and developing appropriate treatments. Recently, it has been shown that cell-cell Notch signaling, one of the major determinants of EC behavior, is regulated by ECM stiffness^6,18^. Here, we showed that this Notch mechanoresponse can be explained by mechanoregulated YAP/TAZ inhibition of both Dll4 and LFng. We then predicted that these YAP/TAZ-Notch interactions can also explain several aspects of the angiogenic dependence on stiffness. Finally, our simulations suggested that Notch activity and angiogenesis can be controlled via cytoskeletal manipulations, as well as by patterns of alternating stiffnesses.

The stiffness-regulated Dll4 expression mediated by YAP/TAZ mechanotransduction has been previously proposed as a responsible factor for the increased Notch activity in EC monolayers on soft substrates^18^. This interaction, however, could not explain the observation that Notch activity decreases for single cells on increasing stiffness^6^. In fact, for single cells, Notch activity is unaffected by changes in intracellular Dll4. Our simulations showed that this single cell response can be explained by a YAP/TAZ-dependent expression of LFng, a hypothesis motivated by previous experiments^29^. Given the role of LFng in increasing Notch-Dll4 affinity, the proposed LFng regulation by YAP/TAZ is also consistent with the increase in trans-endocytosis observed for ECs on soft substrates^6^. Therefore, although we cannot rule out the possible influence of other mechanoregulated Notch processes^17,67^, our computational study points at YAP/TAZ and their regulation of LFng and Dll4 as major determinants of the Notch response to stiffness in ECs. Given the central role of Notch for several EC-regulated processes, ranging from vessel permeability^68,69^ to angiogenesis^19,70^, these mechanisms may play a key role in the functional response of ECs to stiffness, in these contexts too.

Previous experimental studies indeed showed that sprouting angiogenesis is affected by the ECM stiffness. More precisely, there seems to be a positive correlation between sprouting and stiffness for low stiffness regimes^4,71^, while a negative correlation holds beyond a certain stiffness threshold^72^. Our simulations of the tip/stalk phenotype selection suggested that this dependence on stiffness is driven by YAP/TAZ regulation of Dll4 and LFng, which in turn affect the temporal dynamics of tip/stalk selection and filopodia formation (Fig. 3). Specifically, these distinctly predicted filopodia and patterning temporal dynamics translate into sparse network formation and hypersprouting for low and high stiffnesses, respectively^50^, while an optimum physiological vasculature was predicted for intermediate stiffness values. Interestingly, the existence of such an optimal stiffness for vascular formation is also consistent with previous experimental studies^73,74^. In addition to ECM stiffness, other mechanisms influencing YAP/TAZ, such as viscosity^75^ as well as protein density and type^76^, might influence the optimal stiffness level. Increasing the complexity of the model in these directions might therefore provide further insight and more accurate predictions.

The key role of LFng in angiogenesis is also supported by other experimental and computational studies^27,39,52^. In particular, LFng has recently been identified as a temporal regulator of Notch and angiogenesis^27,52^. Additionally, this enzyme and its effects on angiogenesis are increasingly indicated as key factors for arteriovenous malformations, characteristics of hereditary hemorrhagic telangiectasia, resulting from Alk1 gene modifications^52,77^. Our model and simulations, connecting YAP/TAZ to LFng and angiogenesis, might therefore provide additional insight into the role of YAP/TAZ in this pathology, a role that has been recently highlighted^78,79^. The developed model could also be relevant in other disease settings, especially characterized by changes in stiffness, such as cancer^80,81^. ECM stiffening and tumor angiogenesis have been shown to be correlated^82,83^, and targeted therapies addressing angiogenesis have been developed for specific types of cancer^84^. With the suggested interactions between stiffness, the cytoskeleton, YAP/TAZ, and Notch activity, we highlight possible mechanisms determining angiogenesis in these stiffened environments, which might inspire new targets for medical therapies.

In the present study, for example, we explored how the crosstalk between YAP/TAZ and Notch could be employed to control Notch activity and sprouting. Interestingly, we observed that knock down of cytoskeletal elements disrupts YAP/TAZ nuclearization, thereby rescuing Notch activity and tip/stalk pattern formation for high ECM stiffness. These results are consistent with experiments in which ROCK inhibition led to increased vascular density and sprouting of tip cells^85,86^. Nevertheless, severe cytoskeletal manipulations might also affect other mechanisms of angiogenesis, not currently included in the model. For example, myosin activity disruption via Blebbistatin treatment has been shown to affect the sprout structural integrity by impairing adherens-junction organization between tip and stalk cells^87^. Similarly, RhoA inhibition has been shown to affect angiogenesis in a ROCK-independent fashion^86^. Despite these mechanisms not being included in the model yet, our simulations still indicate cytoskeletal modifications as a means to steer angiogenesis, by affecting YAP/TAZ and, in turn, Notch activity and tip/stalk pattern formation.

Finally, the computational results show that another possible avenue to control angiogenesis is via heterogeneous patterns of alternating stiffnesses, which were found to accelerate tip/stalk patterning. The concept that exposure to spatially patterned stimuli can direct sprouting is consistent, for example, with previous experiments showing that VEGF spatial patterns increase vascularization^88^. Similarly, we previously adopted localized Notch ligand patterns to gain control over the spatial location of endothelial sprouts^65^. Via computational simulations, the same study also highlighted the key role of temporal dynamics of Notch signaling for the spatial control of angiogenesis^65^. Our present simulations indicating that heterogeneous stiffness patterns can accelerate tip/stalk pattern formation suggest a new method to control sprouting spatially as well as temporally. Furthermore, they suggest an additional mechanism by which cells themselves might locally control the patterning speed by exerting local forces. Recent experiments have in fact shown that, during sprouting, stromal cells and ECs remodel the ECM giving rise, over time, to very heterogenous stiffness values around sprouting capillaries^89^. These gradually arising heterogeneities of stiffness might contribute to acceleration and stabilization of the tip/stalk patterning via the hypothesized links between stiffness, YAP/TAZ and Notch. This mechanism could act in synergy with random fluctuations in the Notch response, previously shown to affect Notch-mediated patterning in silico^90^ and not yet included in the present model version.

Moreover, our results indicate that the effects of the previously mentioned method featuring alternating lines of coated ligands^65^ can be tuned by the stiffness of the environment. According to the simulations (Fig. 1), the stiffness of the substrates on which the Notch ligands are coated would dictate the resulting local Notch activation and, therefore, the efficacy of these patterns. In particular, performing similar experiments^65^ with relatively soft environments would decrease YAP/TAZ activity, thereby increasing LFng expression and the consequential susceptibility of cells to Notch ligand patterns. Exposing endothelial sprouts to Dll4 patterns coated on soft environments therefore represents a promising research avenue. Additionally, in the future, our computational results concerning the patterning time in response to heterogeneous stiffnesses could be validated by creating gels with stiffness patterns, as previously described^66,91,92^, and by analyzing the spacing and timing of endothelial sprouts^65^.

In conclusion, via computational modeling, we showed the regulation of Dll4 and LFng by YAP/TAZ as a plausible cause for the Notch response to stiffness in both confluent and single ECs. At the functional level, this YAP/TAZ-Notch crosstalk can also explain part of the angiogenic mechanoresponse to ECM stiffness. Finally, our simulations provide promising future research directions, indicating that Notch and angiogenesis might be controlled via cytoskeletal manipulations or patterns of alternating stiffnesses. These findings might have implications for our understanding of angiogenesis-related diseases and open the door to a whole range of new possible engineering interventions.

## Methods

**Fig. 7:**
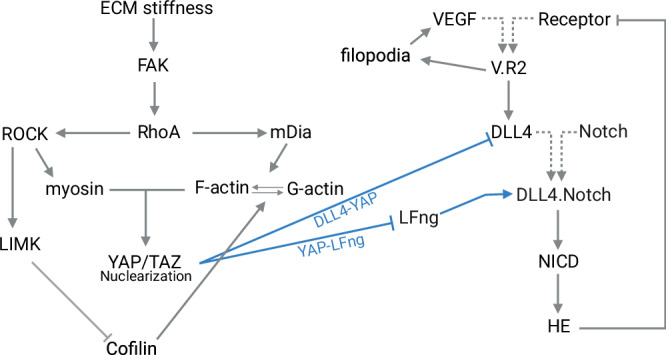
Schematic overview of the interactions described through ODEs in the computational model. In gray, the interactions that were already included in the original models, in blue the interactions added in this study. Dotted arrows indicate formation of a complex rather than activation or upregulation. Created using BioRender.

To model Notch mechanoregulation in ECs, we coupled (Fig. 7) a previously developed ordinary differential equations (ODE) model describing the VEGF-Notch pathway^27,52^ with an ODE model for the stiffness-regulated nuclearization of YAP/TAZ mediated by cellular cytoskeletal elements^37^. These models were coupled through the hypothesized inhibition of Dll4 and LFng by nuclear YAP/TAZ, as supported by previous experiments^18,29^. In what follows, to focus on the novel aspects of the methodology of the present work, the structure of the previous two modeling frameworks is summarized and their new coupling equations are highlighted. Thereafter, the simulations in terms of boundary and initial conditions as well as model parameters are described. For completeness, at the end of the methods, we additionally provide a more detailed description of the previous models at the basis of the present study^27,37,52^.

### Model construction

#### YAP/TAZ model

The YAP/TAZ model, developed by Sun et al.^37^, describes the intracellular signal conversion, from mechanical to biochemical, that occurs through adhesion molecules and cytoskeletal dynamics. Here, the original structure of the model is summarized with protein relationships represented by general functions labeled with $g$, while the complete corresponding ordinary ODEs can be found at the end of the Methods.

In the YAP/TAZ model, ECM stiffness ($E$) induces phosphorylation of FAK ($\varphi$), chosen as a representative adhesion protein regulating downstream signals (Fig. 7). The interactions of FAK with other adhesion proteins are accounted for by considering a non-linear relationship between ECM and FAK phosphorylation, via a second-order Hill function. FAK activation enhances activity of RhoA ($\rho$) (increasing its binding with GTP), in turn leading to actomyosin formation through the activation of both mDia ($\sigma$) and ROCK ($\omega$). On the one hand, mDia is part of the actin polymerization machinery and accelerates the rate of F-actin ($F$) polymerization from G-actin ($G$). On the other hand, ROCK activates LIMK ($L$), which inactivates the F-actin severing effect of cofilin ($C$) and, therefore, also contributes to F-actin stabilization. Additionally, ROCK increases the activity of myosin (Myo, $M$) through phosphorylation. Therefore, as a result of mDia and ROCK activation, myosin and F-actin get activated and form stress fibers. This leads to flattening of the nucleus, allowing for YAP/TAZ ($Y$) nuclearization^36^ (${Y}_{N}$). The computational model can be summarized by the system of equations below, with the inhibitory and activating relationships among the different components represented by functions ${g}_{i}$, with $i$ = 1-9, whose complete definitions can be found at the end of the Methods (Eqs. 13–22). The different proteins associated with the symbols present in the system of equations are described in Table 1:

$$\left\{\begin{array}{c}\frac{d\varphi }{{dt}}={g}_{1}(E,t)\\ \frac{d\rho }{{dt}}={g}_{2}(\varphi ,t)\\ \frac{d\omega }{{dt}}={g}_{3}(\rho ,t)\\ \frac{d\sigma }{{dt}}={g}_{4}(\rho ,t)\\ \frac{{dL}}{{dt}}={g}_{5}(\omega ,t)\\ \frac{{dM}}{{dt}}={g}_{6}(\omega ,t)\\ \frac{{dF}}{{dt}}={g}_{7}(\sigma ,G,C,t)\\ \frac{{dC}}{{dt}}={g}_{8}(L,t)\\ \frac{d{Y}_{N}}{{dt}}={g}_{9}(M,F,t)\end{array}\right.$$

**Table 1:** Symbols used in the YAP/TAZ Eq

FAK	$\varphi$
RhoA	$\rho$
ROCK	$\omega$
mDia	$\sigma$
LIMK	$L$
Myo	$M$
F-actin	$F$
Cofilin	$C$
YAP/TAZ_N_	${Y}_{N}$

#### Notch model

The extended version^52^ of the model developed by Venkatraman^27^ describes the crosstalk between VEGF and Notch signaling in a row of communicating ECs, which determines their filopodia formation and resulting phenotype (tip or stalk). Here, we summarize the assumptions of the model; the corresponding complete ODEs can be found in section “Computational model of VEGF-Notch signaling” (Eqs. 3–12).

When VEGF ($V$) is present, it can bind to the VEGFR ($R$) on ECs (Fig. 7). The consequential VEGFR activation ($V.R$) increases both Dll4 ($D$) expression as well as filopodia ($f$) formation. On the one hand, filopodia formation induces a positive feedback loop, allowing for more VEGF sensing via their extension towards the VEGF source (Fig. 7). Dll4 expression on the other hand, induces a lateral inhibitory effect on neighboring cells. In particular, the expressed Dll4 binds to a Notch ($N$) receptor present on a neighboring cell, forming a Dll4-Notch complex ($D.N$) that leads to Notch cleavage. The resulting cleaved Notch Intracellular Domain (NICD, $I$) translocates to the nucleus where it induces expression of the Notch target genes HES and HEY ($H$), which in turn inhibit VEGFR expression. The computational model can therefore be summarized with the following system of equations, with relationships represented by functions ${g}_{i}$, with $i$ = 10–17. The different proteins associated with the symbols present in the system of equations are described in Table 2. The complete description of the functions ${g}_{i}$ can be found in the last section of the Methods (Eqs. 3–12).

$$\left\{\begin{array}{c}\frac{{dR}}{{dt}}={g}_{10}(V,V.R,H,t)\\ \frac{{dV}.R}{{dt}}={g}_{11}(V,R,t)\\ \frac{{dD}}{{dt}}={g}_{12}(V.R,N,D.N,t)\\ \frac{{dN}}{{dt}}={g}_{13}(D,D.N,t)\\ \frac{{df}}{{dt}}={g}_{14}(V.R,t)\\ \frac{{dD}.N}{{dt}}={g}_{15}(D,N,t)\\ \frac{{dI}}{{dt}}={g}_{16}(D.N,t)\\ \frac{{dH}}{{dt}}={g}_{17}(I,t)\end{array}\right.$$

**Table 2:** Symbols used in the Notch Eq

VEGFR	$R$
VEGF.VEGFR	$V.R$
Dll4	$D$
Notch	$N$
filopodia	$f$
Dll4.Notch	$D.N$
NICD	$I$
Hes1Hey1	$H$

Note that, in the complete model adopted in the present study, the variables $D$, $N$, and $D.N$ are distinguished and distributed among left and right edges of the cells in contact with respective neighbors, considering random motion of unbound Notch proteins across these two edges (see the section “Computational model of VEGF-Notch signaling” for all details).

#### Coupling of the models

The YAP/TAZ and VEGF-Notch models were coupled in this study by including an inhibitory effect of nuclearized YAP/TAZ on the expression of both Dll4 and LFng (Fig. 7). These assumptions were motivated by previous experimental work demonstrating increased Dll4 and LFng expression upon knock down of YAP/TAZ^29,31^, and decreasing Dll4 expression for cells on substrates with increasing stiffness, corresponding to higher YAP/TAZ nuclearization^18^. To implement YAP/TAZ-mediated Dll4 inhibition, inspired by previous work^93^, the Dll4 production term (${D}_{p}$) was inhibited by multiplying it with a Hill-function termed ${X}_{{yt}}$, in our case dependent on nuclear YAP/TAZ (${Y}_{N}$):

1 $${D}_{p}=\left({\beta}_{D}+\frac{\theta * {V\,.\,R}^{2}}{1+{V\,.\,R}^{2}}\right)* {X}_{{yt}};\ \ \ {X}_{{yt}}=\left(\lambda +\frac{1-\lambda }{1+{\left(\frac{{Y}_{N}}{{Y}_{Y0}}\right)}^{n}}\right)$$

Therefore, as in the Venkatraman model^27^, the Dll4 production was determined by adding a baseline protein expression *ß*_*D*_ and the Dll4 production resulting from VEGF-VEGFR complex ($V.R$) activation, as scaled by a parameter $\theta$. This term was multiplied by a function ${X}_{{yt}}$, whose denominator increases with the value of nuclear YAP/TAZ (${Y}_{N}$), thereby causing a decrease in the value of the factor (${X}_{{yt}}$*)* with which ${D}_{p}$ is multiplied. ${Y}_{N}$ is normalized to a baseline value, ${Y}_{Y0}$, from which value onwards we expect to see this inhibitory effect. The effect is scaled through factor $n$, and its maximum effect is determined through $\lambda$ (e.g., $\lambda$ = 0.5 means that for very high ${Y}_{N}$ levels, Dll4 production will be decreased by 50%).

Secondly, we implemented an inhibitory relation between YAP/TAZ and LFng. The regulation of LFng, an enzymatic modulator of binding affinity between Dll4 and Notch, was modeled analogously to previous work^27,52^ by linearly scaling the binding rate of Notch and Dll4 with the concentration of nuclear YAP/TAZ *(Y*_*N*_*):*

2 $${k}_{2{yt}}={k}_{2}* \left(a* {Y}_{N}+b\right)$$

where ${k}_{2{yt}}$ is the Dll4-Notch binding rate as a result of ${Y}_{N}$ activity, whose effect on the reference binding rate ${k}_{2}$ is scaled by parameters $a$ and $b$. Note that parameter $a$ was assigned a negative value, to obtain an inhibitory effect. In summary, the YAP/TAZ and Notch model were coupled through inhibitory YAP/TAZ interactions with Dll4 and LFng (Fig. 7), as hypothesized causes for Notch mechanoregulation.

#### Parameter values

Most parameters were kept at their original values^27,37^, albeit with modifications of FAK-related parameters ($c$, describing the ligand density multiplied with its Young’s modulus at half of the maximal activation rate and $p$, the power) to account for the sensitivity of endothelial YAP/TAZ to stiffness (which was higher than that of the cells in the original model^37^). These FAK-related parameters were calibrated to experimental data^53^ to accommodate for the requirement that YAP/TAZ nuclearization in ECs should exponentially increase already from low stiffness onwards (1.5 kPa), and still gradually increase for stiffnesses of 70 kPa and upwards.

The parameters for the interaction between YAP/TAZ and Dll4 ($\lambda$, ${Y}_{0}$ and $n$ described in Eq. 1) were fitted against previous experimental data showcasing Dll4 expression for cells on substrates of different stiffness, in the presence of VEGF. Again, the main criteria for the fit were that a response should be visible from low stiffnesses onwards (1.5 kPa), and that Dll4 expression should continue to decrease for higher stiffnesses (around 25 kPa), as observed by Matsuo et al.^18^.

For the fit regarding the parameters describing the interaction between YAP/TAZ and LFng ($a$ and $b$) an alternative approach was needed, as no data on LFng expression for intermediate levels of YAP/TAZ nuclearization or substrate stiffness have been reported. In the present study, we hypothesized that YAP/TAZ-LFng is responsible for the Notch mechanoresponse to stiffness when single cells are exposed to substrates with varying stiffnesses coated with Dll4^6^. The YAP/TAZ-LFng interaction was therefore fitted by simulating single cells exposed to Dll4 coating (${D}_{{ext}}$) and comparing Notch reporter gene activity (quantified by $H$ in the model) with previous experimental data^6^. This approach ensured that the interaction to be fitted was not influenced by the YAP/TAZ-Dll4 effects, which cannot play a role for Notch activation in the case of single cells.

${D}_{{ext}}$ needed to accurately reflect the experiments performed by Kretschmer et al.^6^, and was determined by quantitatively tuning ${D}_{{ext}}$ to reach a maximum $H$ increase (normalized against basal production) comparable to the increase observed previously^6^. Graphs showing a comparison of experimental data and the fitted model are shown in Fig. S7. A sensitivity analysis was performed to confirm robustness of our conclusions against these fitted parameters, changed by 10% (Fig. S3).

In simulating interactions with the external environment (e.g., with ${D}_{{ext}}$), no spatial component was considered. We assumed a general level of ${D}_{{ext}}$ and equal exposure for all cells. All fits were conducted using the least squares nonlinear fitting algorithm (lsqnonlin) of MATLAB. For all fits, to find the global minimum across all possible local minima, we ran this fitting algorithm 30 times, with randomly generated starting values (between set boundaries, see Table S3). An overview of all model parameters and corresponding values can be found in the Supplementary Tables S1-2.

Lastly, to simulate a stiffness range from 0.5 kPa to 400 kPa (arbitrarily chosen, to mimic high stiffness) and capture its effect on YAP/TAZ nuclear fractions, we decided to select equal steps in YAP/TAZ nuclear fraction. Starting from 0.5 kPa, 20 equal increments in nuclear YAP/TAZ were taken and the corresponding stiffness was determined, yielding a linear relation between stiffness and nuclearized YAP/TAZ (Fig. S1).

### Model simulations

The coupled computational model was adopted to simulate: (i) the Notch activity of confluent or single cells cultured on 2D substrates with different stiffnesses, with or without stimulation via Dll4 coating; (ii) the process of tip/stalk selection at the onset of sprouting angiogenesis for cells exposed to different ECM stiffnesses; (iii) the potential impact of cytoskeletal interventions on 2D Notch activity and angiogenic sprouting; (iv) and angiogenic sprouting as influenced by spatial variations in substrate stiffness. All simulations were conducted using MATLAB 2021 and the equations were solved using the ODEsolver ode15s, setting the maximum timestep to 100 s. Periodic boundary conditions were implemented in this model, i.e., the first cell interacted with the last cell as if they were connected. At the start of the simulations, concentrations of all intracellular proteins were set to zero. That means, all cells were equal at t = 0 h and there were no initial differences in terms of intracellular protein concentrations. Below, we describe the simulations in more detail.

#### Notch mechanoregulation (in 2D)

2D confluent cell layers were approximated by simulation of a row of cells (with periodic boundary conditions) interacting with each other via Notch signaling, as in previous studies^52,65^. Consistent with experiments^6,18^, a low value of VEGF was adopted for all simulations (${V}_{0}$ = 0.003 cu, cu indicating concentration units) so that cells would not pattern. Experiments with Dll4 coatings were simulated by setting ${D}_{{ext}}$ to 0.426 cu resulting from the calibration (see ‘parameter values’) and setting ${D}_{{ext}}$ to 0 for simulations without a coating. Single cells were simulated by prescribing binding affinity ${k}_{2}$ = 0 for intracellular Dll4 (while binding affinity for extracellular Dll4 was kept at the original ${k}_{2}$ value). In the described simulations, we systematically varied the interactions between YAP/TAZ and Dll4 or LFng through setting $\lambda$ = 1 (to switch off YAP/TAZ inhibiting Dll4) and setting $a$ = 0, $b$ = 1 (to switch off YAP/TAZ inhibiting LFng) to obtain a set of four conditions and analyze their single versus combinatory effects. Finally, we also conducted simulations of cytoskeletal perturbations, as explained in the last section of the Methods.

In all cases, the main measure of interest was the activity of Notch, quantified by NICD. These NICD values were all normalized to 1.5 kPa, the reported baseline value for endothelial tissue^53^. The simulated time period was 24 h, in correspondence with the simulated experiments. All simulations were repeated and averaged for 25 times, to account for possible variability in the outcome.

#### The role of Notch mechanoregulation in patterning stages of angiogenesis

The tip/stalk selection process occurring at the onset of angiogenesis was simulated by exposing the cells to homogeneous stiffnesses and mimicking an acclimatization period of 24 hours in the absence of VEGF (${V}_{0}\,$= 0). Thereafter, the cells were exposed to a VEGF concentration high enough to instigate patterning; ${V}_{0}$ = 0.03 (ten times as high as that used in the Notch mechanoregulation simulations described in the previous section). For all cells, a relatively small random number (order of magnitude 10^−8^) was added to the initial VEGF concentration the cell senses, to account for possible biological variations arising from, for example, differences in cell shape, position and heterogeneous diffusion in the surrounding porous ECM that cause VEGF sensing discrepancies between cells, in addition to correct for the limited precision of MATLAB (16 digits of precision). Again, the effects on Dll4 and LFng were systematically switched on and off through varying $\lambda$, $a$ and $b$. Moreover, we also conducted simulations of cytoskeletal perturbations, as explained in the last Methods’ section.

Each cell was classified as a tip cell if the number of filopodia was larger or equal to the set threshold of 20 filopodia, and as a stalk cell otherwise, in accordance with previous studies^52,63^. Additional simulations were performed with differently chosen thresholds and while absolute values varied, the trends were robust, thereby not affecting the conclusions of the study (Fig. S8). In general, the amounts of filopodia depicted in this study should not be taken absolutely, but rather as a proxy for high or low filopodia activity. A physiological tip/stalk pattern was considered to be achieved when cells exhibited a set of phenotypes consisting of at least 40% tip cells, without any adjacent tip cells (cells at both ends were also considered adjacent). To obtain a proxy for the rate of tip/stalk pattern formation, as in Ristori et al.^52^, the value of 24 h available for cells to pattern upon VEGF exposure was divided by the earliest timepoint for which a physiological tip/stalk pattern was observed (${t}_{p}$) : $\frac{24[h]}{{t}_{p}[h]}$ .

To investigate the effect of heterogeneous stiffness patterns, patterns of 20, 50, 100, and 200 μm of alternating stiffer/softer areas were simulated, mimicking previous experiments^66^. For both stiffnesses, the whole range of selected stiffnesses as described in the section ‘parameter values’ was simulated, yielding 24 × 24 different combinations, all repeated 25 times. A cell was assumed to take up approximately 20/25 μm in these simulations^94^, meaning that the patterns resembled 1, 2, 5 and 9 cells on each stiffness area respectively.

#### Perturbations of cytoskeletal elements

Both for the simulations of 2D layers of cells and the initial patterning stage of angiogenesis, we conducted simulations of perturbations of the cytoskeletal elements. The deactivation rates of Myosin (${k}_{{dmy}}$), ROCK (${k}_{{drock}}$)_,_ and Cofilin (${k}_{{cr}}$) were multiplied with a factor 10 to simulate Blebbistatin, Y-27362 and SZ-3^56,57,95^, while the polymerization rate of F-actin (${k}_{{ra}}$) was multiplied with a factor 1/10 ^37^ to mimic latrunculin^58^. The activator was implemented through multiplication of factor 10 with the baseline activation rate (RhoA, ${k}_{{fkp}}$) to simulate LPA^59^. For completeness, deactivation of RhoA and activation of Myosin, ROCK, F-actin and Cofilin were also simulated by implementation of the opposite factor (i.e., multiplication of 0.1 with deactivation rate ${k}_{{dmy}}$ for example). All simulations were conducted 25 times in case of 2D layers of cell simulations and 50 times in case of patterning, to account for variability in the output resulting from the random factor added to ${V}_{0}$ and the differences in evaluated timesteps by the MATLAB ode15s solver. NICD, LFng and Dll4 expression were normalized to the 1.5 kPa baseline value of the control.

### Computational model of VEGF-Notch signaling

Here, we report the original model of VEGF-Notch signaling in endothelial cells^27,52^, at the basis of the present study. The model uses periodic boundary conditions, distinguishing between the different cell edges. These edges corresponded to ligands and receptors on both the left (subscript ‘L’) and right (subscript ‘R’) edge of the cell. Moreover, the subscript ‘i’ was used to indicate the i^th^ cell in the row, i-1 representing the left neighbor of the cell and i + 1 representing the right neighbor of the cell.

The amount of VEGF (${V}_{i}$) a cell can sense depends on the amount of reference VEGF (${V}_{0}$) (a constant stimulus that the cells are exposed to) and positive feedback by the filopodia (${f}_{i}$)*:*

3 $${V}_{i}={V}_{0}+{k}_{3}{V}_{0}{f}_{i}^{2}$$

scaled by parameter ${k}_{3}$. This VEGF can bind to the VEGF receptor (${R}_{i}$) thereby establishing a VEGF-VEGFR complex (${V.R}_{i}$) leading to upregulation of filopodia formation. The amount of ${R}_{i}$ available for binding with VEGF is downregulated by Hes1/Hey1 (${H}_{i}$) leading to the following equations:

4 $$\frac{{{dR}}_{i}}{{dt}}={-k}_{1}{V}_{i}{R}_{i}+{k}_{-1}{V.R}_{i}-\phi {R}_{i}+\gamma -{k}_{{inh}}{R}_{i}{H}_{i}^{2}$$

5 $$\frac{{{dV}.R}_{i}}{{dt}}={k}_{1}{V}_{i}{R}_{i}-{k}_{-1}{V.R}_{i}-\phi {V.R}_{i}$$

6 $$\frac{{{df}}_{i}}{{dt}}=\beta +{k}_{f}{V.R}_{i}^{2}-{k}_{-f}{f}_{i}$$

where ${k}_{1}$ and ${k}_{-1}$ represent the association of ${V.R}_{i}$ and disassociation of ${V.R}_{i}$ respectively, $\phi$ and $\gamma$ indicate protein degradation and production, and ${k}_{{inh}}$ scales the impact of inhibition by Hes1/Hey1. Regarding the filopodia, ${k}_{-f}$ and ${k}_{f}$ represent the filopodia turnover rate and filopodia formation resulting from the upregulation by ${V.R}_{i}$ respectively, whereas $\beta$ represents basal filopodia formation. Activation of the VEGF receptor through ${V.R}_{i}$, yields upregulation of Dll4 (${D}_{i}$) which can then bind to Notch (${N}_{i}$) leading to complex formation (${D.N}_{i}$) and subsequential activation of Notch. Alternatively, Notch can be activated through external Dll4 (${D}_{{ext}}$) also leading to complex formation (${D}_{{ext}}.{N}_{i}$). This leads to the following equations (now visualized on the left edge of the cell, but this is analogous for the right edge):

7 $$\begin{array}{l}\frac{{{dD}}_{{L}_{i}}}{{dt}}=\frac{1}{2}\left(\beta +\theta \frac{{V.R}_{i}^{2}}{1+{V.R}_{i}^{2}}\right){X}_{{yt}}-{k}_{{2}_{{yt}}}{D}_{{L}_{i}}{N}_{{R}_{i-1}}+{k}_{-2}{D.N}_{{R}_{i-1}}\\\qquad\quad\,+W\left(\frac{{D}_{{L}_{i}}+{D}_{{R}_{i}}}{2}-{D}_{{L}_{i}}\right)-\phi {D}_{{L}_{i}}\end{array}$$

8 $$\begin{array}{l}\frac{{{dN}}_{{L}_{i}}}{{dt}}=\frac{\gamma }{2}-{k}_{{2}_{{yt}}}{N}_{{L}_{i}}({D}_{{R}_{i-1}}+{D}_{{ext}})+{k}_{-2}\left({D\,\cdot\,N}_{{L}_{i}}+{D}_{{ext}}\,\cdot\,{N}_{{L}_{i}}\right)\\\qquad\,+W\left(\frac{{N}_{{L}_{i}}+{N}_{{R}_{i}}}{2}-{N}_{{L}_{i}}\right)-\phi {N}_{{L}_{i}}\end{array}$$

9 $$\frac{{D.N}_{{L}_{i}}}{{dt}}={k}_{{2}_{{yt}}}{N}_{{L}_{i}}{D}_{{R}_{i-1}}-{k}_{-2}{D.N}_{{L}_{i}}-{k}_{{cat}}{D.N}_{{L}_{i}}-\phi {D.N}_{{L}_{i}}$$

10 $$\frac{{D}_{{ext}}.{N}_{{L}_{i}}}{{dt}}={k}_{{2}_{{yt}}}{N}_{{L}_{i}}{D}_{{ext}}-{k}_{-2}{D}_{{ext}}.{N}_{{L}_{i}}-{k}_{{cat}}{D}_{{ext}}.{N}_{{L}_{i}}-\phi {D}_{{ext}}.{N}_{{L}_{i}}$$

where *θ* represents the factor of downregulation of Dll4 production by ${V.R}_{i}$, ${k}_{{2}_{{yt}}}$ and ${k}_{-2}$ represent the rates of association and dissociation of the ${D.N}_{i}$ complex, $W$ indicates diffusion of unbound Notch across both cell edges and ${k}_{{cat}}$ indicates catalysis of the ${D.N}_{i}$ complex. The factors ${X}_{{yt}}$ and ${k}_{{2}_{{yt}}}$, which were added in the present study and were not present in the original version of the model, represent the inhibitory interactions between the YAP/TAZ and Notch pathway, as described in Eqs. 1–2. Finally, upon Notch activation, the Notch Intracellular Domain, NICD (${I}_{i}$), increases which in turn leads to Hes1/Hey1 expression through:

11 $$\frac{{{dI}}_{i}}{{dt}}={k}_{{cat}}\left({D.N}_{{L}_{i}}+{D.N}_{{R}_{i}}+{D}_{{ext}}.{N}_{{L}_{i}}+{D}_{{ext}}.{N}_{{R}_{i}}\right)-\phi {I}_{i}$$

12 $$\frac{d{H}_{i}}{{dt}}=\beta +\theta \frac{{I}_{i}^{2}}{1+{I}_{i}^{2}}-\phi {H}_{i}$$

### Computational model of YAP/TAZ signaling

Here, we report the complete model of YAP/TAZ signaling^37^ at the basis of the present study, with adjustments to accommodate the simulation of the activity in endothelial cells. Stiffness sensation occurs through focal adhesion kinase, FAK ($\varphi$) which can get phosphorylated (${\varphi }_{p}$) as a result of integrin and protein clustering; modeled here via Michaelis-Menten kinetics. The power (0.5) and the parameter $c$ were changed with respect to the original model^37^, to better fit the EC response to stiffness. Resulting from the activation of such adhesion molecules, RhoA ($\rho$) can bind to GTP, which in turn drives actomyosin formation through the activation of ROCK ($\omega$) and mDia ($\sigma$) described by the following equations:

13 $$\frac{{d\varphi}_{p}}{{dt}}={k}_{{sfdf}}\left(\frac{{({d}_{l}E)}^{0.5}}{{{c}^{0.5}+({d}_{l}E)}^{0.5}}\right)({{\varphi }_{0}}-{{\varphi}_{p}})$$

14 $$\frac{d\rho }{{dt}}={k}_{{fkp}}\left(\upsilon {\varphi }_{p}^{2}+1\right)\left({\rho }_{0}-\rho \right)-{k}_{{dp}}\rho$$

15 $$\frac{d\omega }{{dt}}={k}_{{rp}}\rho \left({\omega }_{0}-\omega \right)-{k}_{{drock}}\omega$$

16 $$\frac{d\sigma }{{dt}}={k}_{{mp}}\rho \left({\sigma }_{0}-\sigma \right)-{k}_{{dmdia}}\sigma$$

in which ${k}_{{sfdf}}$ describes the net activation/dephosphorylation rate of $\varphi$, ${d}_{l}$ and $E$ describe properties of the ECM, ligand density and stiffness of the ECM, respectively. $c$, a component of the Michaelis Menten kinetics formulation, describes the ${d}_{l}$*$E$ value for which the activation rate of $\varphi$ is half of its maximum and ${\varphi }_{0}$ represents the total amount of FAK (which does not change). Similarly, ${\rho }_{0}$, ${\omega }_{0}$ and ${\sigma }_{0}$ represent the total amounts of RhoA, ROCK and mDia. The baseline activation rate by FAK is represented by $\upsilon$ and activation through other mechanisms by ${k}_{{fkp}}$, while ${k}_{{dp}}$ represents the deactivation rate. For ROCK and mDia, the activation rates by RhoA are described by ${k}_{{rp}}$ and ${k}_{{mp}}$ respectively, whereas the deactivation rates are represented by ${k}_{{drock}}$ and ${k}_{{dmdia}}$. Activated ROCK in turn promotes activity of LIM kinase, LIMK ($L$) as well as that of myosin ($M$) by phosphorylation of its kinase and inhibition of phosphatase, described by:

17 $$\frac{{dM}}{{dt}}={k}_{{mr}}(\varepsilon T+1)\left({M}_{0}-M\right)-{k}_{{dmy}}M$$

18 $$\frac{{dL}}{{dt}}={k}_{{lr}}\left(\tau T+1\right)\left({L}_{0}-L\right)-{k}_{{dl}}L$$

for which ${M}_{0}$ and ${L}_{0}$ represent the total amounts of Myosin and LIMK, ${k}_{{mr}}$ and ${k}_{{lr}}$ indicate the activation by other pathways than ROCK while ${k}_{{dmy}}$ and ${k}_{{dl}}$ indicate their deactivation. Both myosin and LIMK are also activated and amplified through ROCK, here scaled by $\varepsilon$ and $\tau$. The interaction with ROCK is modeled through a smoothing function ($T$) thereby mimicking the requirement that the ROCK concentration needs to exceed a threshold in order to activate. This was implemented by usage of the build-in error function (${erf}$) in MATLAB, through:

19 $$T=\frac{{e}^{-{S}_{\varphi }^{2}{(\varphi -{\varphi }_{S})}^{2}}}{2\sqrt{\pi }{S}_{\varphi }}+\frac{\varphi -{\varphi }_{S}}{2}(1+{\rm{erf}}({S}_{\varphi }(\varphi -{\varphi }_{S}))$$

for which the activator, in this case ROCK, has an element-specific activation threshold (${\varphi }_{s}$) as well as a parameter specific to the specific smoothing function (${S}_{\varphi }$) determining the sharpness of the response. In turn, LIMK inactivates cofilin ($C$) a protein severing F-actin ($F$) by phosphorylation. F-actin is not only affected by cofilin, but also through mDia, which polymerizes G-actin to F-actin, equated by:

20 $$\frac{{dC}}{{dt}}={k}_{{to}}\left({C}_{0}-C\right)-{k}_{{cr}}(1-{k}_{{ll}}{l}_{0}){L}^{2}C$$

21 $$\frac{{dF}}{{dt}}={k}_{{ra}}\left(\alpha T+1\right)({F}_{0}-F)-{k}_{{dep}}F-{k}_{{fc}1}{CF}$$

in which ${C}_{0}$ and ${F}_{0}$ indicate total amounts of cofilin and F-actin, ${k}_{{to}}$ and ${k}_{{cr}}$ represent the dephosphorylation and phosphorylation rates of cofilin respectively, ${k}_{{cr}}$ resulting from LIMK, while ${k}_{{ll}}$ represents phosphorylation inhibition by LIMK resulting from the amount of LATS_0_, ${l}_{0}$, present in the cell. ${k}_{{ra}}$ represents the polymerization rate of cytoplasmic F-actin while ${k}_{{dep}}$ represents its depolymerization rate and ${k}_{{fc}1}$ represents its disassembly rate by cofilin. $\alpha$ represents the amplification of polymerization due to mDia, incorporated using the smoothing function, $T$. Finally, activated myosin and F-actin form stress fibers, leading to flattening of the nucleus allowing for YAP/TAZ ($Y$) nuclearization (${Y}_{N}$)^36^, described by:

22 $$\frac{d{Y}_{N}}{{dt}}=\left({k}_{{cn}}+{k}_{{cy}}{FM}\right)\left({Y}_{0}-{Y}_{N}\right)-({k}_{{nc}}+{k}_{{ly}}{l}_{p}){Y}_{N}$$

in which translocation rates from the cytoplasm to the nucleus independent and dependent of cytoplasmic F-actin and myosin are described by ${k}_{{cn}}$ and ${k}_{{cy}}$ respectively, whereas translocation from the nucleus to the cytoplasm without active LATS and with active LATS (${l}_{p}$) are described by ${k}_{{nc}}$ and ${k}_{{ly}}$ respectively. Lastly, ${Y}_{0}$ represents the total amount of YAP/TAZ.

## Supplementary information

Supplementary material

## Data Availability

All data will be available at 4TU. ResearchData, through: 10.4121/e12cc475-432a-4315-bc64-15a8888e3571.

## Code availability

All computational code will be available at 4TU. ResearchData, through: 10.4121/e12cc475-432a-4315-bc64-15a8888e3571.
