# Supplementary material for: YAP/TAZ drives Notch and angiogenesis mechanoregulation in silico
Source: NPJ Syst Biol Appl. 2024 Oct 5;10:116. doi: 10.1038/s41540-024-00444-3 (PMC11455968; doi:10.1038/s41540-024-00444-3)
Supplement: Supplementary file 1 — Supplementary material [file 41540_2024_444_MOESM1_ESM.docx]

**Supplementary Information**

**Table S1. Parameter values**

| **Param.** | **Value** | **Description** |
| --- | --- | --- |
| $V_{0}$ | 0.3 / 0.03 / 0 [cu] | Reference amount of VEGF, depending on the scenario |
| $k_{3}$ | 0.005 [cu^-2^] | Factor scaling positive feedback between filopodia and VEGF |
| $k_{1}$ | 0.1 [cu^-1^ sec^-1^] | Rate of association of V.R |
| $k_{-1}$ | 0.001 [sec^-1^] | Rate of disassociation of V.R |
| $\phi$ | 0.005 [sec^-1^] | Protein degradation |
| $\gamma$ | 0.005 [cu sec^-1^] | Protein production |
| $k_{inh}$ | 0.005 [cu^-2^ sec^-1^] | Scales the impact of inhibition by H |
| $\beta$ | 0.001 [cu sec^-1^] | Basal filopodia formation |
| $k_{f}$ | 0.1 [sec^-1^] | Filopodia formation rate |
| $k_{-f}$ | 0.001 [sec^-1^] | Rate of filopodia turnover |
| $\theta$ | 0.1[sec^-1^] | Downregulation rate of Dll4 production by V.R |
| $k_{-2}$ | 0.1 [sec^-1^] | Disassociation rate of D.N |
| $W$ | 0.001 [sec^-1^] | Diffusion of unbound Notch across both cell edges |
| $k_{cat}$ | 0.1 [sec^-1^] | Catalysis rate of D.N |
| $D_{exdt}$ | 0.426 / 0 [cu] | External Dll4 (from a Dll4 coating), depending on the scenario |
| $k_{2}$ | 0.002 [cu^-1^ sec^-1^] | Association rate of D and N |
| $k_{sfdf}$ | 0.225 [] | Net activation/dephosphorylation rate of K |
| $d_{l}$ | 1 [] | Relative ligand density in ECM |
| $\upsilon$ | 500 [] | Activation rate of RhoA, by FAK |
| $k_{fkp}$ | 0.018 [sec^-1^] | Activation rate of RhoA through other mechanisms than FAK |
| $k_{dp}$ | 0.625 [sec^-1^] | Deactivation rate |
| $k_{rp}$ | 2.2 [sec^-1^] | Activation rate of ROCK by RhoA |
| $k_{mp}$ | 1 [sec^-1^] | Activation rate of mDia by RhoA |
| $k_{drock}$ | 0.8 [sec^-1^] | Deactivation rate of ROCK |
| $k_{dmdia}$ | 1 [sec^-1^] | Deactivation rate of mDia |
| $k_{mr}$ | 0.015 [sec^-1^] | Activation of Myo by other pathways than ROCK |
| $k_{lr}$ | 0.07 [sec^-1^] | Activation of LIMK by other pathways than ROCK |
| $k_{dmy}$ | 0.067 [sec^-1^] | Deactivation of Myo |
| $k_{dl}$ | 2 [sec^-1^] | Deactivation of LIMK |
| $\varepsilon$ | 40 [] | Activation of Myo through ROCK |
| $\tau$ | 200 [] | Activation of LIMK through ROCK |
| $S_{\varphi}$ | 13 [] | Smoothing parameter, determining sharpness of the activation – ROCK |
| $\varphi_{s}$ | 0.26 [] | Activation threshold for the smoothing function - ROCK |
| $S_{\sigma}$ | 10 [] | Smoothing parameter, determining sharpness of the activation – mDia |
| $\sigma_{S}$ | 0.13 [] | Activation threshold for the smoothing function - mDia |
| $k_{to}$ | 0.04 [sec^-1^] | Dephosphorylation rate of cofilin |
| $k_{cr}$ | 0.7 [sec^-1^] | Rate of phosphorylation of cofilin by LIMK |
| $k_{ll}$ | 0.8 [sec^-1^] | Phosphorylation inhibition rate of cofilin by LIMK, resulting from LATS_0_ |
| $k_{ra}$ | 0.4 [sec^-1^] | Polymerization rate of cytoplasmic F-actin |
| $k_{dep}$ | 0.35 [sec^-1^] | Depolymerization rate of cytoplasmic F-actin |
| $k_{fc1}$ | 8 [sec^-1^] | Disassembly rate of F-actin by cofilin |
| $\alpha$ | 40 [] | Amplification of polymerization of F-actin due to mDia |
| $k_{cn}$ | 0.1 [sec^-1^] | YAP/TAZ nuclear translocation rate independent of F-actin |
| $k_{cy}$ | 20 [sec^-1^] | YAP/TAZ nuclear translocation rate dependent on F-actin |
| $k_{nc}$ | 3 [sec^-1^] | YAP/TAZ cytoplasmic translocation rate independent of LATS_0_ |
| $k_{ly}$ | 6 [sec^-1^] | YAP/TAZ cytoplasmic translocation rate dependent on LATS_0_ |
| $l_{0}$ | 0.5 | Total amount of LATS, LATS_0_ |
| $l_{p}$ | 0.05 | Phosphorylated LATS |
| $\varphi_{0}$*,* $\rho_{0}$*,* $\omega_{0}$*,* $\sigma_{0}$*,* $M_{0}$*,* $L_{0}$*,* $C_{0}$*,* $F_{0}$*,* $Y_{0}$ | 1 | Total amounts of available protein |

“cu” stands for concentration units.

**Table S2.** The parameters that were altered/added in the development of the model:

| **Param.** | **Value** | **Description** |
| --- | --- | --- |
| $\lambda$ | 0.1 [] | Maximum inhibitory effect of $Y_{N}$ on Dll4 production |
| $Y_{Y0}$ | 0.47 [cu] | Fraction of $Y_{N}$ from which onwards $Y$ starts inhibiting Dll4 |
| $n$ | 6.5 [] | Scaling the effect of YAP/TAZ inhibition of Dll4 |
| $a$ | -0.6121 [] | Scaling the effect of YAP/TAZ inhibition of LFng |
| $b$ | 1.0578 [] | Scaling the effect of YAP/TAZ inhibition of LFng |
| $c$ | 7.5 [kPa] | $d_{l}*E$ value for which activation rate equals half the maximum activation rate |

**Table S3. Boundaries used for the fitting procedure**

| **Param.** | **Lower limit** | **Upper limit** | **Argumentation** |
| --- | --- | --- | --- |
| $\lambda$ | 0 | 1 | The effect of the used Hill function can be between 0 (0) and 100% (1) |
| $Y_{Y0}$ | 0 | 1 | The fraction of nuclear YAP/TAZ can be between 0 and 1. |
| $n$ | 0.01 | 10 | If this parameter is equal to 0, the effect of $Y_{Y0}$would be negated, if it is equal to values above 10 there is only an initial response to stiffness, and very little response to higher stiffnesses. |
|  |  |  |  |
| $a$ | -15 | -0.01 | If this parameter is equal to 0, the effect of nuclear YAP/TAZ on LFng would be negated, if it is equal to values above 15 there is only an initial response to stiffness, and very little response to higher stiffnesses. |
| $b$ | 0.01 | 15 | If this parameter is equal to 0, there would not be any scaling of the YAP/TAZ-LFng interaction. Upper value was chosen to align with the upper value of $a$. |

**Supplementary Figure S1**


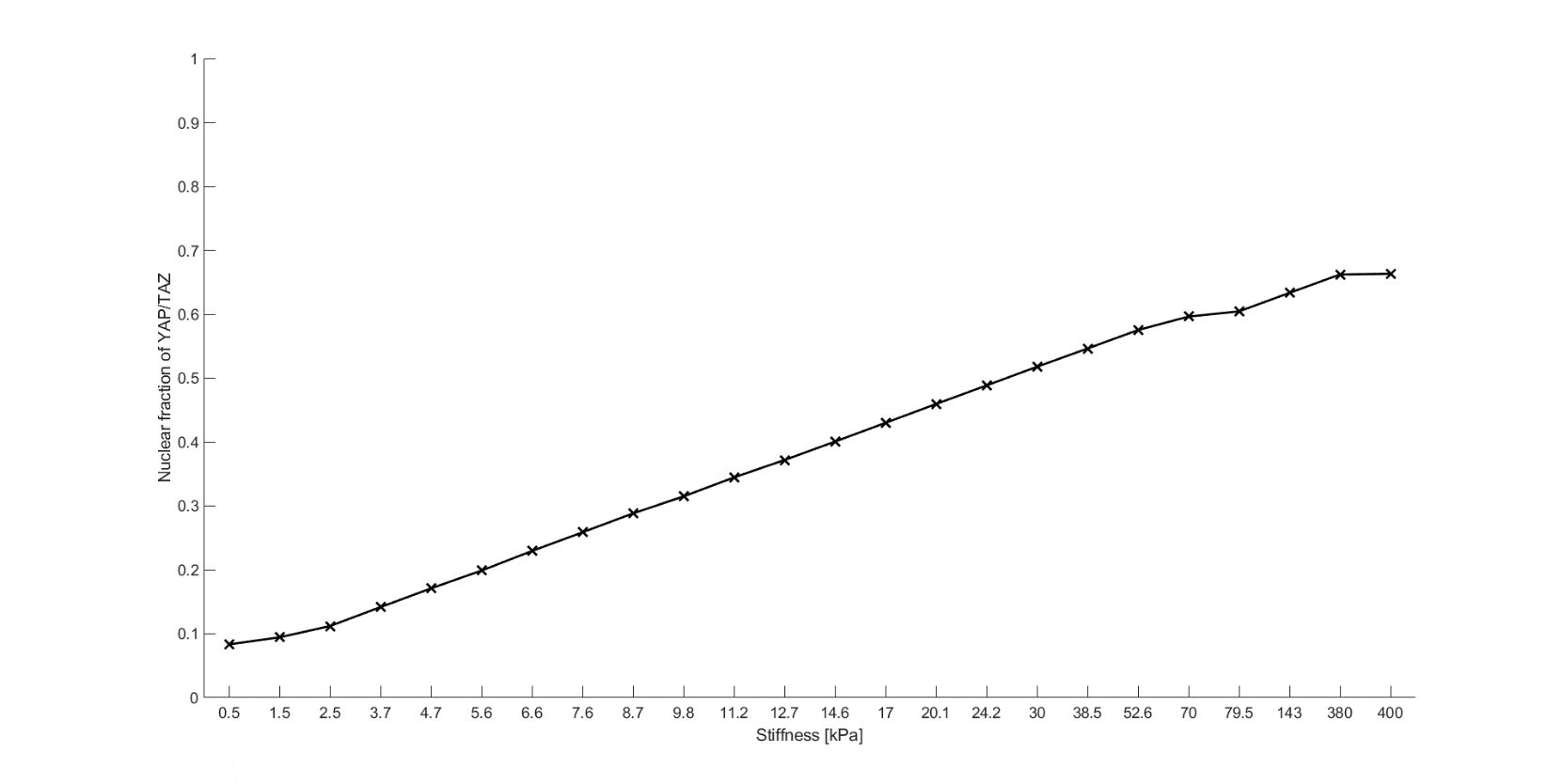


*Figure S1: Stiffness-values (x-axis) that were used in the simulations, to obtain a linear increase in nuclear fraction of YAP/TAZ (y-axis). The curve is not completely linear, as four additional stiffness-values were included, to allow for comparison with experimental data: 0.5, 1.5, 70 and 400 kPa.*

**Supplementary Figure S2**


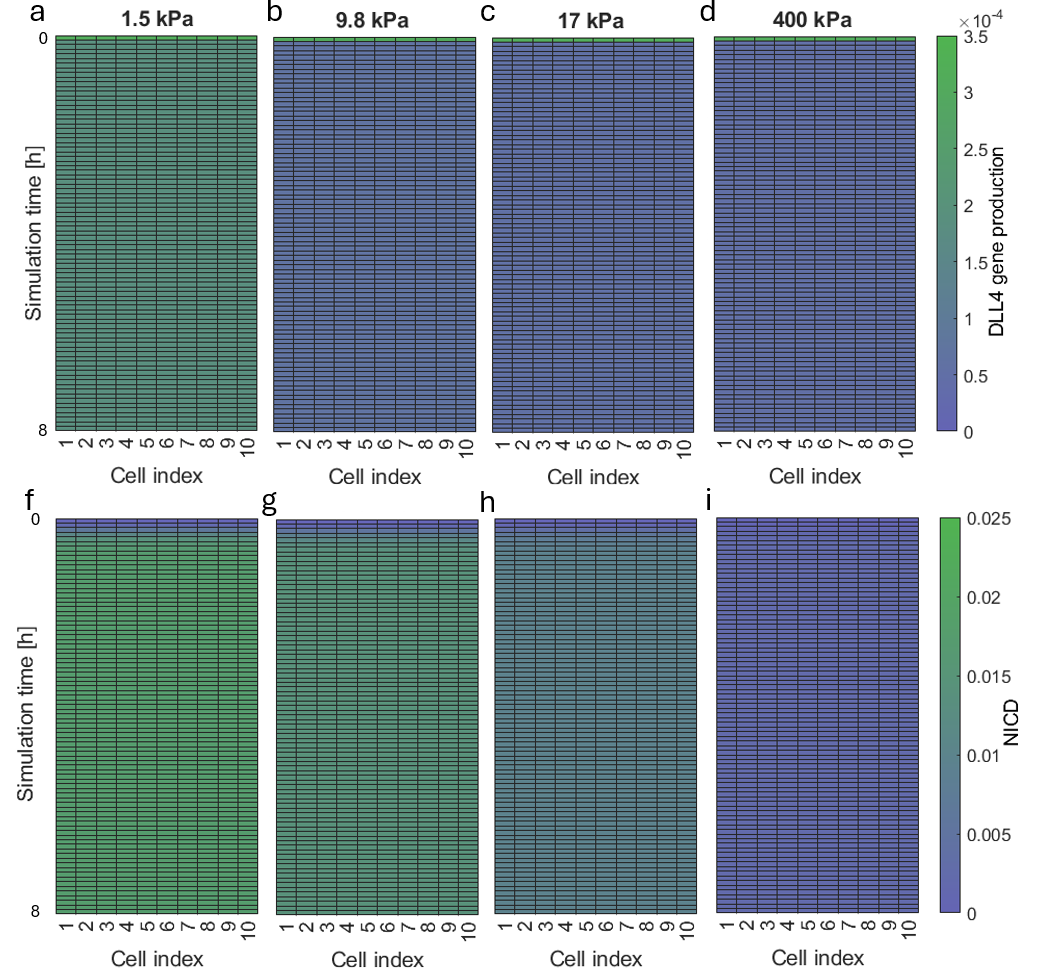


*Figure S2: Homogeneous Dll4 and NICD distributions are predicted for cells cultured for 24h on substrates with different stiffness, without VEGF stimulation. The predicted values decrease for increasing stiffness. The numbers on the horizontal axes represent the cell number, such that the colors of each column above those numbers represent the values of Dll4 expression (****a-d****) or NICD (****e-f****) for that specific cell. Subfigures* ***a*** *and* ***e*** *show simulation results for 1.5 kPa;* ***b*** *and* ***f*** *show simulation results for 9.8 kPa;* ***c*** *and* ***g*** *show simulation results for 17 kPa; and* ***d*** *and* ***h*** *show simulation results for 400 kPa.*

**Supplementary Figure S3**


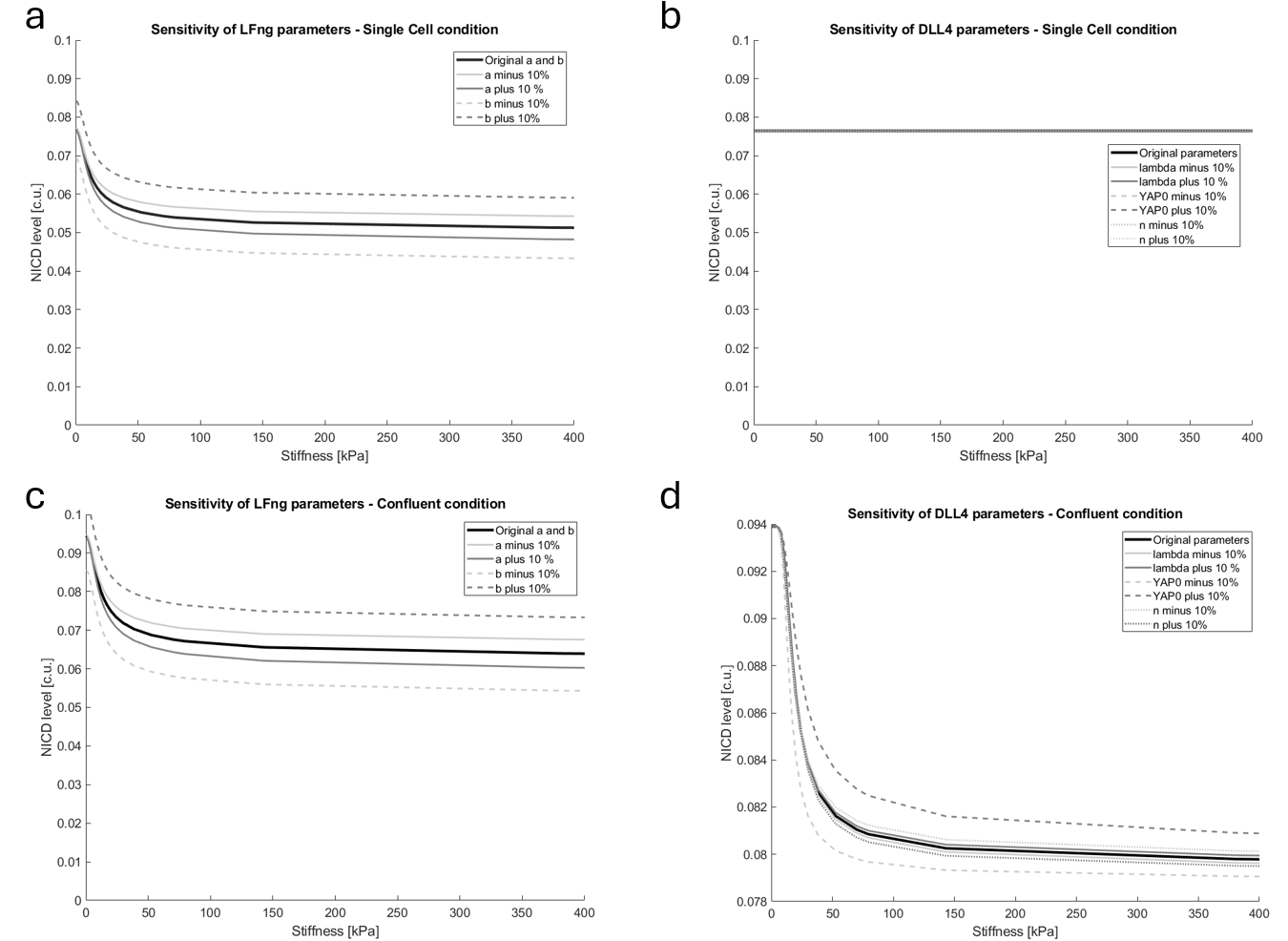


*Figure S3: Sensitivity analysis performed to investigate the sensitivity of model output to fitted model parameters.* ***a*** *and* ***b*** *show simulation results for the single cell condition with activating Dll4 coating, while* ***c*** *and* ***d*** *show the simulation results for the confluent condition, with Dll4 coating.* ***a*** *and* ***c*** *show the results for the model including just the YAP/TAZ-LFng interaction, while* ***b*** *and* ***d*** *show the results for the model including just the YAP/TAZ-Dll4 interaction. In all cases, the black graphs indicate model output with original parameter settings, while the lightest graphs indicate a reduction of 10% of the respective parameter values and the intermediately grey graphs indicate an increase of 10% of the respective parameter values.*

**Supplementary Figure S4**


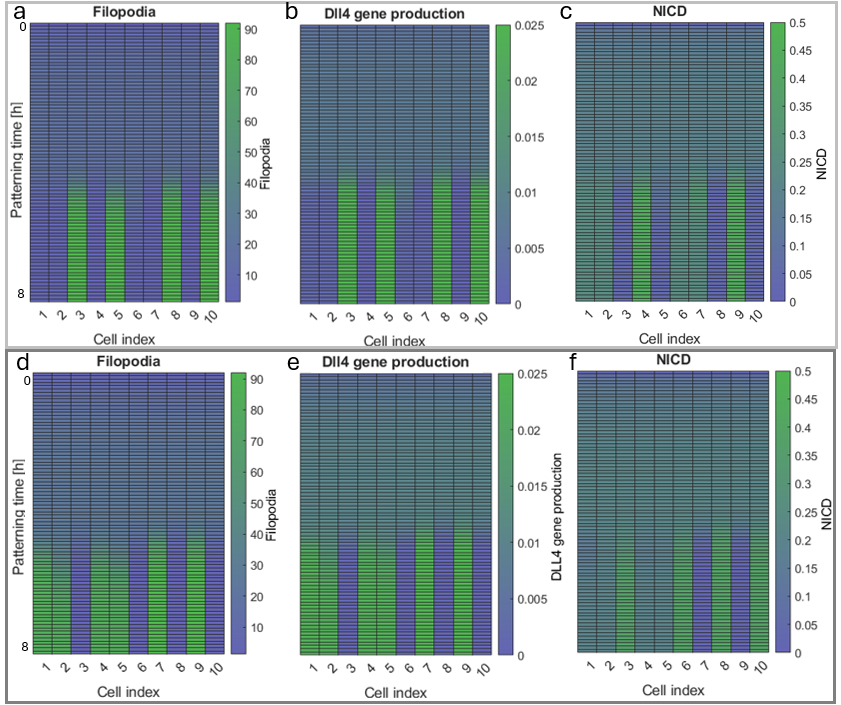


*Figure S4: Examples of simulations predicting two adjacent stalk or tip cells. (****a****-****c****): Patterning results for a simulation that yielded 2 adjacent stalk cells, resulting in 40% tip cells. As shown in subpanel* ***c****, this arrangement leads to intermediate levels of NICD for the adjacent stalk cells compared to the high NICD level obtained for non-adjacent stalk cells. This intermediate value results from intracellular Notch heterogeneity: one side of the cell exhibits high Notch and the other side exhibits low Notch. (****d****-****f****): Patterning results for a simulation that yielded 2 adjacent tip cells, resulting in 60% tip cells. In subpanel* ***e*** *it is clear that all tip cells have high Dll4 production, while the NICD levels are again at an intermediate level for the adjacent cells (****f****).*

**Supplementary Figure S5**


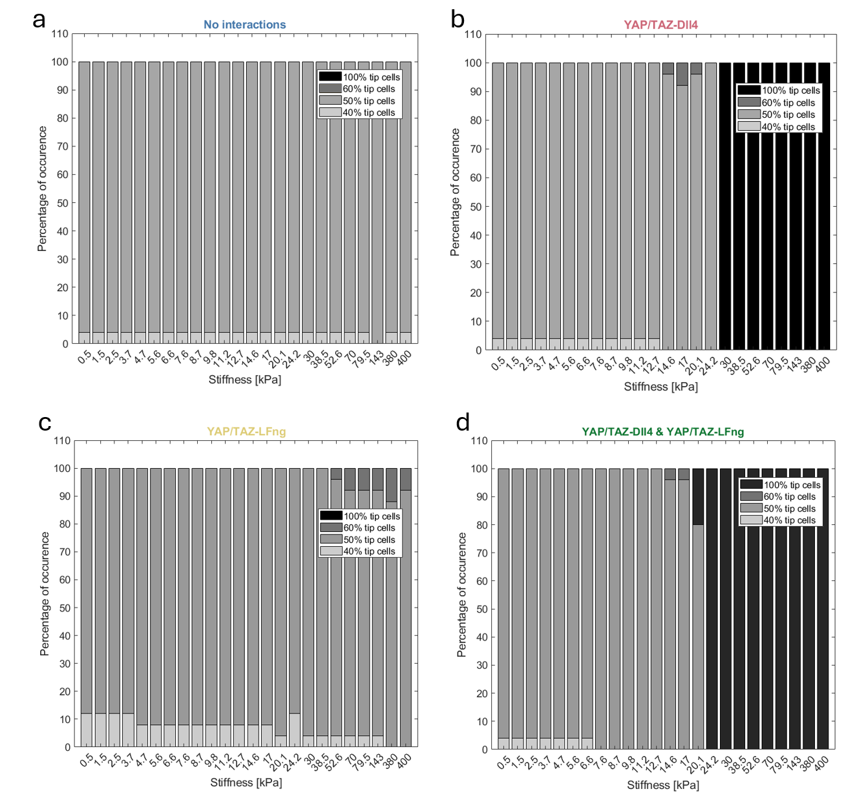


*Figure S5: Increased tip cell percentages are predicted for increasing values of ECM stiffness. The reported tip cell percentages correspond to the simulation results shown in Fig. 3, obtained by simulating cells exposed to VEGF for 24h, running the simulations 25 times. The panels respectively correspond to the model without YAP/TAZ-Notch interactions (****a****), the model including the YAP/TAZ-Dll4 interaction (****b****), the model including the YAP/TAZ-LFng interaction (****c****), and the full model including both interactions (****d****). The darker the color depicted in the stacked bar plot, the higher the corresponding percentage of tip cells, as shown in the legend.*

**Supplementary Figure S6**


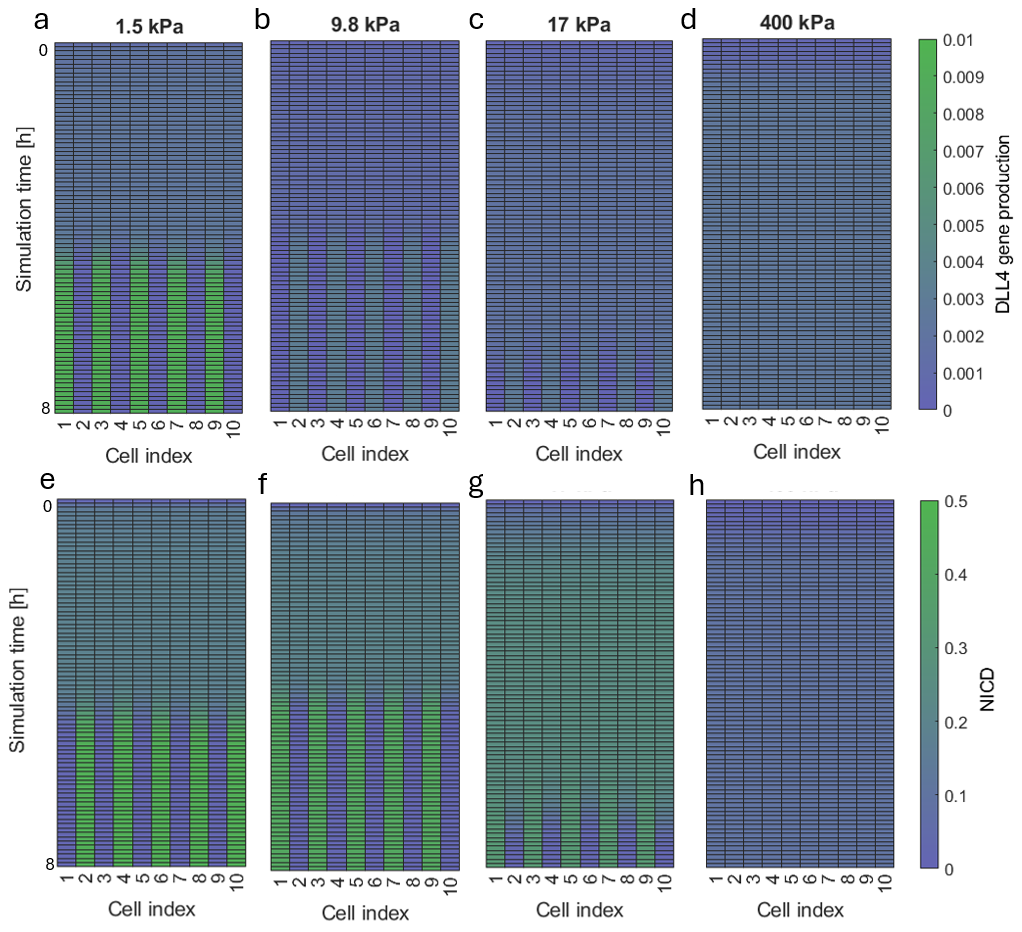


*Figure S6: When cells were exposed to VEGF to initiate patterning after 24 hours, high Dll4 production in combination with low NICD levels was predicted for tip cells, while the opposite held for stalk cells, which showed low Dll4 production and high NICD levels in simulations (48 hours). The final 24 hours were visualized here, showing pattern formation for all stiffnesses up until 17 kPa (****a****,* ***b****,* ***c****,* ***e****,* ***f*** *and* ***g****). The predicted average values of Dll4 and NICD decrease for increasing stiffness. The numbers on the horizontal axis represent the cell number, such that the colors of each column above those numbers represent the values of Dll4 expression (****a****-****d****) or NICD levels (****e****-****h****) for that specific cell. Subfigures* ***a*** *and* ***e*** *show simulation results for 1.5 kPa;* ***b*** *and* ***f*** *show simulation results for 9.8 kPa;* ***c*** *and* ***g*** *show simulation results for 17 kPa; and* ***d*** *and* ***h*** *show simulation results for 400 kPa.*

**Supplementary Figure S7**


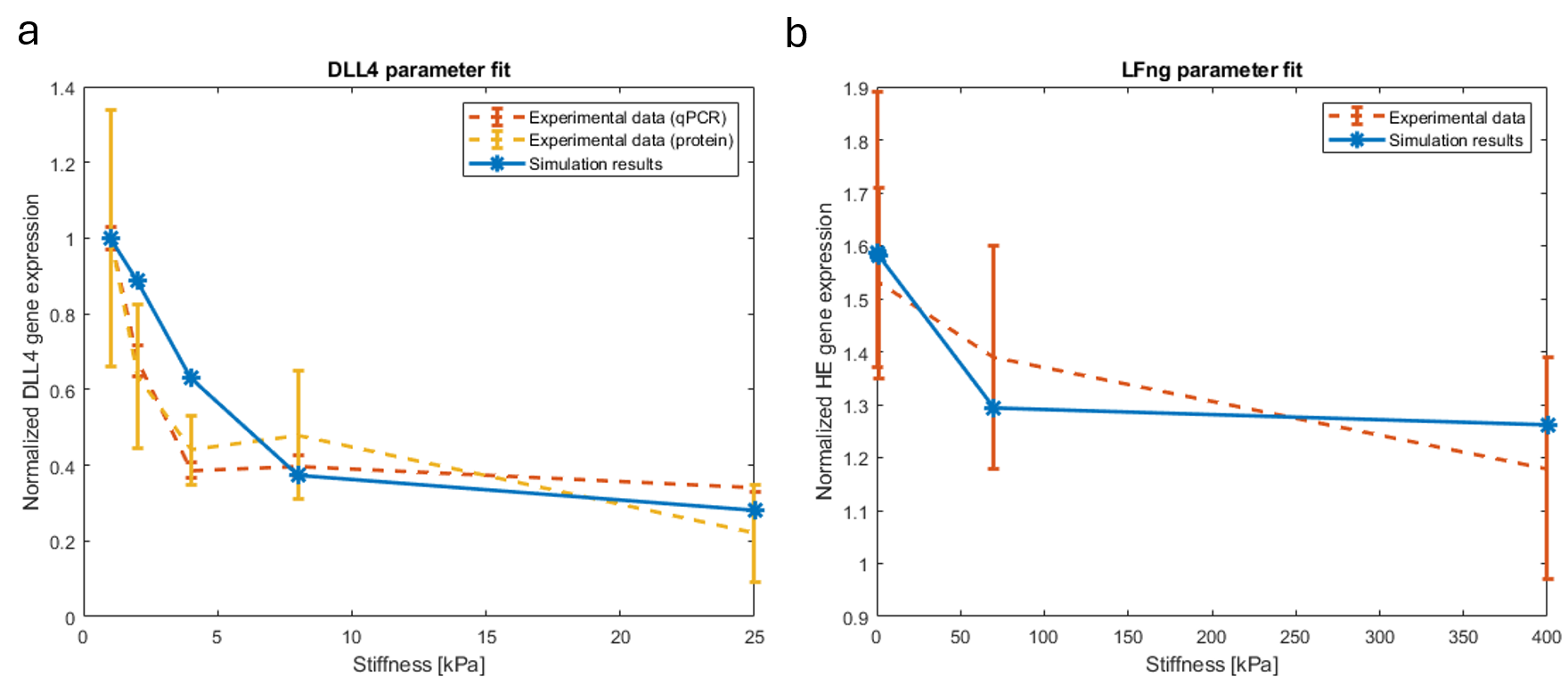


*Figure S7: Comparison between the fitted model and previous experimental data (Matsuo et al.^18^ and Kretschmer et al.^6^). On the left (****a****), the fits for the parameters of the YAP/TAZ-Dll4 interaction are shown. On the right (****b****), the fits for the parameters of the YAP/TAZ-LFng interaction are represented. For both cases, the continuous line connects the values predicted by the fitted simulations, while the dashed lines connect the values and standard deviations of the single experimental results.*

**Supplementary Figure S8**


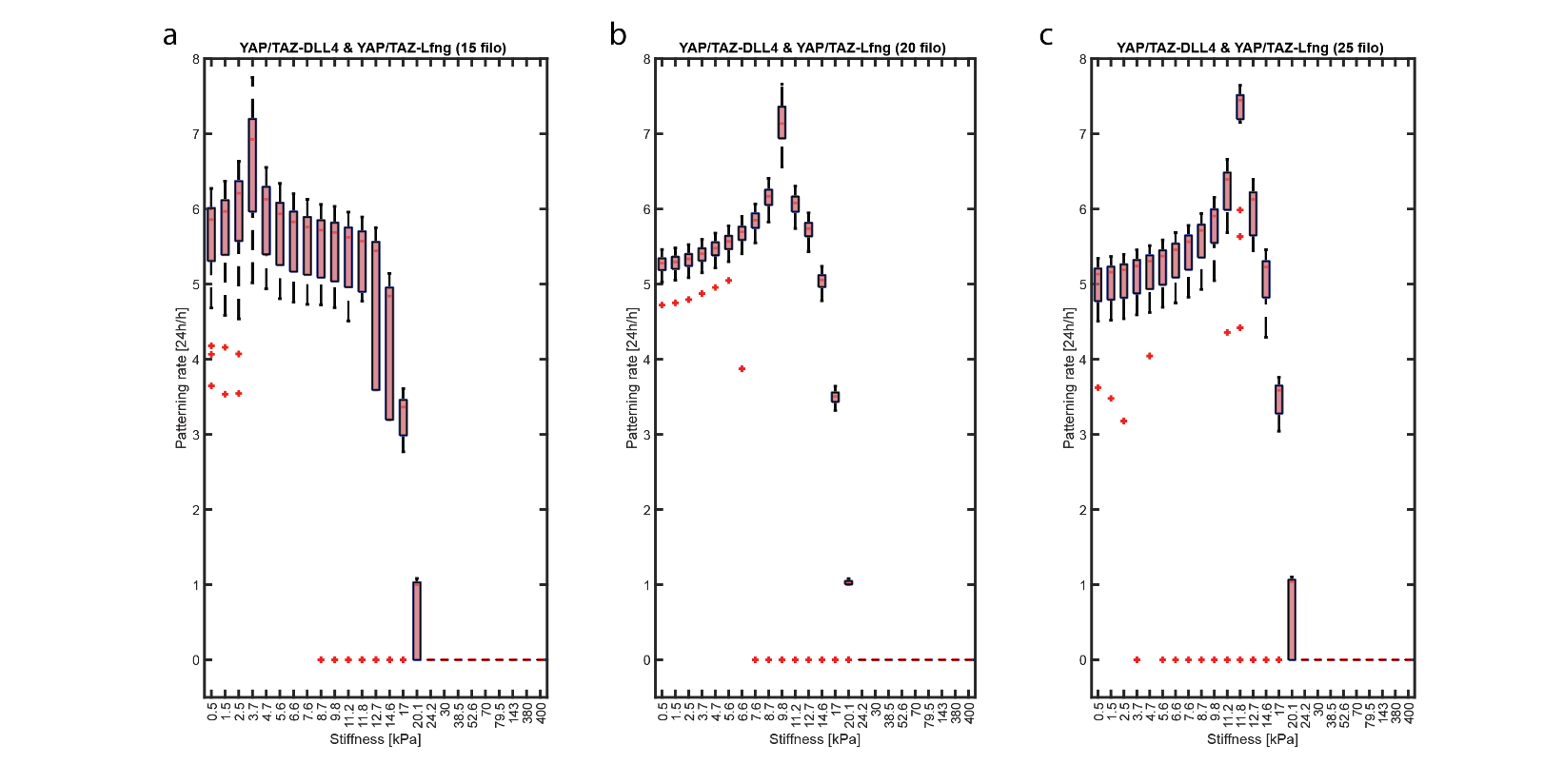


*Figure S8: The biphasic response in the patterning rate is robust. Repetition of Fig. 3g; simulation of a row of 10 cells, after an acclimatization period of 24 hours (no exposure to VEGF) followed by 24 hours of exposure to VEGF to instigate patterning. Patterning rates were determined by dividing the possible 24 hours of patterning by the patterning time. All simulations were repeated 25 times, and the associated data are represented such that the boxes depict quartiles 2 and 3, the red line inside the box depicts the median, the whiskers indicate the min- and maximum and the red plusses indicate the outliers* ***b*** *shows the original model settings, i.e. a threshold of 20 filopodia in order for a cell to be classified as a tip cell, whereas* ***a*** *shows simulations results for a threshold of 15 filopodia and,* ***c*** *shows the simulation results for a threshold of 25 filopodia. Trends remain the same, although the stiffness for which the patterning optimum occurs shifts in correspondence with the shift in filopodia threshold; i.e. increasing this threshold leads to an increase in optimal stiffness, while decreasing it leads to a decrease in optimal stiffness.*
